# Triazine herbicide prometryn alters epoxide hydrolase activity and increases cytochrome P450 metabolites in murine livers via lipidomic profiling

**DOI:** 10.1038/s41598-024-69557-3

**Published:** 2024-08-19

**Authors:** Rasheed O. Sule, Christophe Morisseau, Jun Yang, Bruce D. Hammock, Aldrin V. Gomes

**Affiliations:** 1 Department of Neurobiology, Physiology, and Behavior, University of California, Davis, One Shields Ave, Davis, CA 95616 USA; 2 Department of Entomology and Nematology, University of California, Davis, Davis, CA 95616 USA; 3 Comprehensive Cancer Center, University of California, Davis, Davis, CA 95616 USA; 4 Department of Physiology and Membrane Biology, University of California, Davis, Davis, CA 95616 USA; 5 Center for Mitochondrial and Epigenomic Medicine, Children’s Hospital of Philadelphia, Philadelphia, PA 19104 USA; 6 Department of Pathology and Laboratory Medicine, Children’s Hospital of Philadelphia, Philadelphia, PA 19104 USA

**Keywords:** Prometryn, Lipidomics, Oxylipins, Oxidative stress, Inflammation, Liver, Epoxide hydrolase, Metabolites, Pesticides, Arachidonic acid (AA), Epoxyeicosatrienoic acid (EpETrE), Eicosapentaenoic acid (EPA), Docosahexaenoic acid (DHA), Epoxyeicosatetraenoic acid (EpETE), Epoxydocosapentaenoic acid (EpDPE), Proteomic analysis, Lipidomics

## Abstract

Oxylipins are a group of bioactive fatty acid metabolites generated via enzymatic oxygenation. They are notably involved in inflammation, pain, vascular tone, hemostasis, thrombosis, immunity, and coagulation. Oxylipins have become the focus of therapeutic intervention since they are implicated in many conditions, such as nonalcoholic fatty liver disease, cardiovascular disease, and aging. The liver plays a crucial role in lipid metabolism and distribution throughout the organism. Long-term exposure to pesticides is suspected to contribute to hepatic carcinogenesis via notable disruption of lipid metabolism. Prometryn is a methylthio-s-triazine herbicide used to control the growth of annual broadleaf and grass weeds in many cultivated plants. The amounts of prometryn documented in the environment, mainly waters, soil and plants used for human and domestic consumption are significantly high. Previous research revealed that prometryn decreased liver development during zebrafish embryogenesis. To understand the mechanisms by which prometryn could induce hepatotoxicity, the effect of prometryn (185 mg/kg every 48 h for seven days) was investigated on hepatic and plasma oxylipin levels in mice. Using an unbiased LC–MS/MS-based lipidomics approach, prometryn was found to alter oxylipins metabolites that are mainly derived from cytochrome P450 (CYP) and lipoxygenase (LOX) in both mice liver and plasma. Lipidomic analysis revealed that the hepatotoxic effects of prometryn are associated with increased epoxide hydrolase (EH) products, increased sEH and mEH enzymatic activities, and induction of oxidative stress. Furthermore, 9-HODE and 13-HODE levels were significantly increased in prometryn treated mice liver, suggesting increased levels of oxidation products. Together, these results support that sEH may be an important component of pesticide-induced liver toxicity.

## Introduction

Oxygenated lipids are commonly referred as oxylipins. They belong to a group of molecules derived from the oxygenation of polyunsaturated fatty acids (PUFAs) that can mediate molecular signaling, including inflammatory pathways, maintenance of redox, metabolic and protein homeostasis^1,2^. They serve a variety of important signaling roles within the cell^1^. Upon cell activation, the formation of oxylipins begins when fatty acids are released from membrane phospholipids by cytosolic phospholipase A2 (cPLA2)^3^. In the absence of cPLA2, oxylipins may be formed via an alternative route, such as through triacylglycerol lipase, as observed in mast cells^3,4^. When fatty acids are released by cPLA2, they are converted to oxylipins via three main pathways: cyclooxygenase (COX), lipoxygenase (LOX), and cytochrome P-450 (CYP) activities^3^. PUFAs such as arachidonic acid (AA), eicosapentaenoic acid (EPA), docosahexaenoic acid (DHA), linoleic acid (LA), and α-linoleic acid (ALA) are metabolized into biologically active signaling molecules by undergoing further processing by CYP, LOX, and COX-mediated pathways^5^. Because of their role in cardiovascular disease and age-related degeneration, oxylipins are being recognized as viable targets for these diseases^3^.

Although advantageous, the excessive use and misuse of pesticides may pose adverse effects, such as bioaccumulation in the environment and non-target organisms toxicity^6,7^. Pesticides enter surface and groundwater primarily as run-off from agricultural fields and industrial wastewater^8^. The direct and indirect contamination of surface waters by pesticides occur via aerial drift, watershed run-off, rainfall erosion, soil leeching, and accidental spillage during the widespread use of these chemicals in agriculture and forestry^9^. 2,4-Bis (isopropylamino)-6-methylthio-1,3,5-triazine (prometryn) belongs to the triazine herbicide classification, and it is used to control weeds in cotton, pigeon peas, celery, and dill, and is currently contained in seventeen registered products^7^. Prometryn also eliminates aquatic weeds and hazardous algae in the aquaculture industry^10^. It is classified as a persistent organic compound since it resists biological and chemical degradation^11^. In fact, prometryn is highly stable in the environment, with long half-lives in soil (316 days) and water (70 days), making it a severe ecosystem pollutant^12^.

Moreover, prometryn has been detected at a concentration of 3–6.1 μg/L in different rivers and lakes in Europe^7,12^. Previous studies highlighted that high amount of triazine herbicides were detected in groundwater, surface waters, river basins in agricultural areas in Europe and North America^7,13,14^. Residential proximity to agricultural pesticide applications is an important source of ambient environmental exposure, where pesticides applied from the air and ground may drift from intended sites thereby posing non-target organism toxicity, such as humans^15^.

Pesticides are metabolized in the liver due to the presence of numerous metabolizing enzymes. Long-term exposure to pesticides has been hypothesized to play a role in the progression of liver carcinogenesis via alteration to mechanisms involved in cell adhesion, oxidative stress, genotoxicity, tumor promotion, immunotoxicity, and hormonal action^15^. It appears that exposure to dichlorodiphenyltrichloroethane (DDT), an organochlorine insecticide widely used in the mid-twentieth century, and its metabolite, dichlorodiphenyldichloroethylene (DDE) led to the development of hepatocellular carcinoma (HCC) or primary liver cancer in rodents^15–18^. Similarly, prometryn exposure caused deleterious defects in liver development during embryogenesis in zebrafish thereby implicating its role in possibly being hepatotoxic^6^.

Oxylipins are produced in various tissues, including liver, white adipose tissue, kidney, and ileum^19^. Since the liver plays an important role in xenobiotics metabolism, we postulated that prometryn alteration in oxylipin metabolites contributes to its liver toxicity. However, the mechanism and signaling pathways by which prometryn affects the liver is unknown. There is also no information readily available in the literature on its effect on oxylipins. In this study, liquid chromatography tandem mass spectrometry (LC–MS/MS)-based lipidomics approach was used to analyze oxylipins produced by COX, LOX, and CYP enzymes from multiple PUFA substrates to determine the effects of prometryn exposure in mice liver.

## Materials and methods

### Cell culture

HepG2 liver carcinoma cell line obtained from the ATTC (Manassas, VA, USA) were maintained in high glucose Dulbecco's modified Eagle's medium (DMEM, Gibco) supplemented with 2 mM l-glutamine, 110 mg/L sodium pyruvate, 100 U/mL penicillin, 100 μg/mL streptomycin and 10% fetal bovine serum (FBS). Cells were maintained in a humidified atmosphere containing 5% CO_2_ at 37 °C.

### Intracellular reactive oxygen species (ROS) detection

The cell-permeant reagent 2′,7′-dichlorodihydrofluorescein diacetate (H_2_DCFDA), a fluorogenic dye (Enzo Life Sciences, Inc., Farmingdale, NY) was utilized to study the intracellular production of ROS in HepG2 cells. Briefly, 1 × 10^4^ cells/well of HepG2 liver cells were plated on a 96-well black plate and incubated overnight. The cells were washed twice with PBS, and then treated with 10 μM of H_2_DCFDA in phenol red free media for 30 min at 37 °C. Following incubation, the wells were washed again with PBS, and the cells were treated with different concentrations (10, 20, 30 μM) of prometryn and 100 μM H_2_O_2_ (positive control) in phenol free media. ROS production was determined by measuring the fluorescence at Ex/Em at 502/523 nm^20^.

CellROX Deep Red (Invitrogen Detection Technologies, ThermoFisher Scientific) was also used to detect oxidative stress in HepG2 liver carcinoma cells. CellROX Deep Red Reagent is cell-permeable DNA dye that is non-fluorescent or very weakly fluorescent while in a reduced state. Upon oxidation, CellROX Deep Red exhibits strong fluorogenic signal localized primarily in the cytoplasm. Briefly, 1 × 10^4^ cells/well of HepG2 cells were seeded on a 96-well black plate and incubated overnight. The cells were treated with 5 μM of CellROX Deep Red for 30 min at 37 °C. Following incubation, the wells were washed three times with PBS, and the cells were treated with 10–50 μM of prometryn, 100 μM H_2_O_2_ (positive control), or pretreated 1 h ahead with 10 μM MitoTEMPO then prometryn was added. ROS production was determined by measuring the fluorescence at Ex/Em at 644/665 nm.

### Animal studies

The animal experiment was performed in accordance with the protocols approved by the Institutional Animal Care and Use Committee (IACUC) of the University of California, Davis. This study is reported in accordance with the ARRIVE guidelines (https://arriveguidelines.org). C57BL/6J male mice (10 weeks old) were used for the study. Mice were maintained at controlled temperature and humidity and had free access to food and water^21^. The control mice received pure corn oil subcutaneously every 48 h for 7 days. The prometryn treatment group mice were subcutaneously treated with 185 mg/kg of prometryn dissolved in pure corn oil every 48 h for 7 days, at 100 μL per animal.

Corn oil was used to solubilize the prometryn. The dose of prometryn given to mice is approximately 1/20 of the LD_50 (mice)_ = 3750 mg/kg as previously described^22^. The amount of prometryn used was the lowest dose previously used in three publications that utilized this compound in a mouse model^22–24^. None of the animals showed any clinical signs of disease or behavior disorders; at the end of the experiment, all animals were alive. Their body weight was measured at the beginning and end of the experiment, and no differences were observed between the control and treated groups (data not shown). The mice were euthanized using 1.5% isoflurane, and livers were immediately excised and quickly washed twice in ice-cold PBS and then preserved in liquid nitrogen. Simultaneously, blood was collected from the mice and centrifuged for 10 min to collect the plasma. Prior to use, the liver tissue was individually pulverized with a cryogenic-based metal tissue mill and stored at − 80 °C until use for lipidomics and biochemical assays.

### LC–MS/MS-based lipidomic profiling of oxylipins

#### Extraction and analyses of the regulatory lipid mediator

The extraction process is similar to the protocol as described in previously published papers^25–27^. To extract lipid metabolites from liver tissues, approximately 50 μL of plasma and 100 mg of liver tissues were mixed with an antioxidant solution (0.2 mg/mL butylated hydroxytoluene and 0.2 mg/mL triphenylphosphine in methanol), 10 µL of methanol containing deuterated internal standards (500 nM of d4-6-keto PGF1a, d4-TXB2, d4-PGE2, d4-LTB4, d11-14,15-DiHETrE, d6-20-HETE, d4-9-HODE, d8-5-HETE, d11-11,12-EET) (Cayman Chemical, Ann Arbor, MI). Afterwards, 400 μL of cold methanol solution containing 0.1% acetic acid and 0.2 mg/mL butylated hydroxytoluene, BHT (Sigma-Aldrich, St. Louis, MO) was added to the liver tissue samples and stored at − 80 °C for 30 min. After freezing, samples were homogenized using Retsch MM301 ball mills (Retsch Gmbh, Germany) at 30 Hz for 10 min and the resulting homogenates were kept at − 80 °C overnight. The homogenates were centrifuged at 16,000*g* for 10 min, the supernatants were collected, and remaining pellets were washed with 100 µL of ice-cold methanol containing 0.1% acetic acid and 0.1% butylated hydroxytoluene (BHT) and centrifuged at 16,000*g* for 10 min. The supernatants of each sample were combined and diluted with 2 mL of H_2_O and loaded onto Waters Oasis HLB cartridges (Waters, Milford, MA) solid phase extraction (SPE) cartridges.

#### Measurement

The oxylipins amounts were measured on a 1200 SL ultra-high-performance liquid chromatography (UHPLC) (Agilent, Santa Clara, CA) interfaced with a 4000 QTRAP mass spectrometer (SCIEX, Redwood City, CA). The separation conditions for LC were optimized to separate the critical pairs of oxylipins, which share the same multiple reaction monitoring (MRM) transitions. In brief, separation was achieved on an Agilent Eclipse Plus C18 150 × 2.1 mm 1.8 μm column with mobile phases of water with 0.1% acetic acid as mobile phase A and acetonitrile/methanol (84/16) with 0.1% acetic acid as mobile phase B. All the parameters on the mass spectrometer were optimized using pure standards (Cayman Chemical, Ann Arbor, MI) under negative mode. A scheduled MRM scan mode was employed to increase the sensitivity of the measurement. The mass spectrum peak areas of all detected oxylipins were integrated after the metabolite mass spectrometry data of different samples were obtained, and the same metabolite mass spectrum peaks within the different samples were integrated for correction. The concentrations of the lipid metabolites were calculated and normalized against the calibration curve with standards. The standards was used to control for extraction efficiency, evaluate LC–MS performance, and normalize the LC–MS data.

### Radiometric assay of mEH and sEH activity

Pulverized liver samples (50 mg) were suspended in chilled sodium phosphate buffer (20 mM pH 7.4) containing 5 mM ethylenediaminetetraacetic acid (EDTA), protease inhibitor (1 mM PMSF) and 1 mM DTT. The mixtures were strongly mixed for at least 10 s, and centrifuged at 1000*g* for 20 min at 4 °C. The supernatant was collected and used as enzyme source. It was then frozen at − 80 °C until usage. The supernatant protein concentration was quantified using the bicinchoninic acid (BCA) method using bovine serum albumin (BSA) as standard.

#### mEH activity

The microsomal epoxide hydrolase (mEH) activity was determined using [^3^H]-*cis*-stilbene oxide (c-SO) as substrate^28^. The extract from the liver tissue was diluted 45-folds in Tris/HCl buffer (0.1 M, pH 9.0) containing 0.1 mg/mL BSA. The reaction was started by adding 1 µL of a 5 mM solution of c-SO in ethanol to 100 µL of diluted extracts ([S]_final_ = 50 µM)^21^. The mixture was incubated at 37 °C for 5–30 min. The reaction was then quenched by adding 250 μL of isooctane, which extracts the remaining epoxide from the aqueous phase. Extractions with 1-hexanol were performed in parallel to assess the possible presence of glutathione transferase activity which could also transform the substrate. The activity was followed by measuring the quantity of radioactive diol formed in the aqueous phase using a scintillation counter. Assays were performed in triplicate.

#### sEH activity

The soluble epoxide hydrolase (sEH) activity was determined using [^3^H]-*trans*-diphenyl-propene oxide (t-DPPO) as substrate^29^. The extract from the liver tissue was diluted 300-folds in sodium phosphate buffer (0.1 M, pH 7.4) containing 0.1 mg/mL BSA. Briefly, 1 µL of a 5 mM solution of t-DPPO in DMSO was added to 100 µL of the tissue extracts ([S]_final_ = 50 µM)^21^. The mixture was incubated at 37 °C for 5–20 min, and the reaction was quenched by addition of 60 µL of methanol and 200 µL of isooctane, which extracts the remaining epoxide from the aqueous phase. Extractions of an identical reaction with 1-hexanol were performed in parallel to assess the possible presence of glutathione transferase activity, which could also transform the substrate. Hexanol extracts epoxides and diols into the upper phase while glutathione conjugates remain in the lower water phase. The activity was followed by measuring the quantity of radioactive diol formed in the aqueous phase using a scintillation counter (TriCarb 2810 TR, Perkin Elmer, Shelton, CT). Assays were performed in triplicate.

### Lipid peroxidation

Lipid peroxidation assay was carried out using Sigma Aldrich Lipid Peroxidation (MDA) Assay Kit (Cat. # MAK085) according to the manufacturer's instructions. The assay principle is determined by the reaction of malondialdehyde (MDA) with thiobarbituric acid (TBA) to form a colorimetric/fluorometric product that is proportional to the MDA present in the sample. Briefly, approximately 15 mg of liver tissue samples were homogenized in 300 μL of ice-cold MDA lysis buffer containing 3 μL of BHT. The samples were centrifuged at 13,000*g* for 10 min at 4 °C to remove insoluble material. The supernatant from the homogenized liver sample and MDA standards provided in the kit were added to the wells followed by the addition of TBA solution. The samples and MDA standards were incubated at 95 °C for 60 min on a heat metal block. Afterwards, they were cooled to RT in an ice bath for 10 min. The absorbance was measured at 532 nm using a microplate reader. Assays were performed in triplicate.

### Hydrogen peroxide (H_2_O_2_) assay

Hydrogen peroxide activity assay was carried out as described previously^30^. The assay was performed using Sigma Aldrich Fluorometric Hydrogen Peroxide Assay Kit (Cat. # MAK165). All steps were carried out according to the manufacturer's instructions. The assay principle is based on the peroxidase substrate that reacts with H_2_O_2_ to produce an infra-red fluorescent product. Approximately 15 mg of liver tissue samples were homogenized in 250 μL of ice cold 1× PBS buffer. The samples were centrifuged at 10,000*g* for 10 min at 4 °C. The supernatant was collected and diluted to similar concentrations using the kit's assay buffer. The supernatant and H_2_O_2_ standards were added to the wells followed by the addition of a master mix that contained infra-red peroxidase substrate, 20 units/mL peroxidase, and assay buffer. The plate was incubated for 15 min at room temperature and protected from light. The fluorescence intensity was measured at excitation/emission = 540/590 nm. Assays were performed in triplicate.

### 26S proteasome activity assay

26S proteasome activity assay was carried out as described previously^31,32^. Briefly, 20 mg of pulverized liver tissue samples were homogenized in 26S proteasome lysis buffer (50 mM Tris, 150 mM sodium chloride (NaCl), 1 mM ethylenediaminetetraacetic acid (EDTA), 5 mM magnesium chloride (MgCl_2_), 1 mM DTT (freshly added) [pH 7.5]) with a hand-held Potter–Elvehjem homogenizer. The homogenates were centrifuged at 12,000*g* for 15 min at 4 °C and the supernatant collected. The protein concentration was quantified and diluted with the lysis buffer to 2 μg/μL concentration. The AMC conjugated activity-specific peptides were used to assess the different proteasome catalytic subunit activities, β2 (trypsin-like), β5 (chymotrypsin-like), and β1 (caspase-like), by adding 20 µg of protein diluted in 26S proteasome lysis buffer and 100 μM ATP. In order to control for non-proteasomal-mediated cleavage of substrates, a specific proteasome inhibitor: 10 μM bortezomib (β5 activity) and 100 μM bortezomib (β1 and β2 activities) was used in some wells while dimethyl sulfoxide (DMSO) was used in wells without the proteasome inhibitor^20^. Samples were incubated with inhibitors at RT for 20 min, protected from direct light exposure. Then, specific fluorescence-tagged AMC peptides for each proteasomal β subunits, 100 μM Z-LLE-AMC for β1, 100 μM Boc-LSTR-AMC for β2, and 100 μM Suc-LLVY-AMC for β5, were used to initiate the reaction. Proteasomal activity was assessed by continuous fluorescent measurement of the released AMC at 37 °C for 2 h, using a fluorometric plate reader (Tecan Infinite M1000 Pro) at an excitation wavelength of 390 nm and an emission wavelength of 460 nm.

### Immunoproteasome activity assay

Immunoproteasome (β5i and β1i) activity assay was performed as previously described^32^. 20 mg of liver tissue samples were homogenized in immunoproteasome buffer (50 mM Tris, 5 mM MgCl_2_, 20 mM potassium chloride (KCl), 1 mM DTT (freshly added), pH 7.5) with a hand-held Potter–Elvehjem homogenizer. The homogenates were centrifuged at 12,000*g* for 15 min at 4 °C, and the supernatant was collected. 20 μg of protein were incubated with immunoproteasome buffer and a specific immunoproteasome inhibitor or an equivalent volume of DMSO at RT for 20 min protected from direct light exposure. The specific inhibitors for β5i 20 µM ONX-0914 (Abmole Bioscience Inc., Houston, TX, Cat. No. M2071) and for β1i 50 µM bortezomib were used to control for non-immunoproteasome-mediated cleavage of substrates and evaluate the specificity of the assay^30^. 25 µM fluorogenic substrates Ac-ANW-2R110 (AAT Bioquest, Inc, CA) for β5i and Ac-PAL-2R110 (AAT Bioquest, Inc, CA) for β1i were used to initiate the reaction. The fluorescence intensity was measured every 5 min for 60 min at an excitation of 498 nm and an emission of 520 nm at 37 °C in a Tecan Infinite M1000 Pro fluorometer.

### Immunoblotting

#### Sample preparation

Pulverized liver tissue (20 mg) was homogenized in ice-cold 1× lysis buffer (50 mM Tris, 150 mM NaCl, 1 mM EDTA, 5 mM MgCl_2_) [pH 7.5]) using a glass Dounce homogenizer. The homogenates were centrifuged at 12,000*g* for 15 min at 4 °C, and the supernatant was collected. Protein concentrations were determined in triplicate using the BCA protein assay (Bio-Rad, Hercules, CA, Cat. #500-0119) and were diluted to equal protein concentrations. Heart samples were mixed with 4× Laemmli sample buffer (8% SDS, 40% glycerol, 0.4% bromophenol blue, 240 mM Tris, pH 6.8) with freshly added β-mercaptoethanol and the samples were denatured at 95 °C for 5 min.

#### Electrophoresis and western blotting

Equal amounts of protein (20 µg per lane) were separated on 4–20% TGX precast gels (Cat. # 567-1094, Bio-Rad) for 80 min at 120 V. Proteins were transferred to a nitrocellulose membrane (Trans-Blot Turbo Midi Nitrocellulose, #170-4159, Bio-Rad) using the Trans-Blot Turbo Transfer System (Cat # 170-4155, Bio-Rad)^20^. The membranes were then stained with Ponceau S and imaged to serve as a loading control for total protein normalization of Western blots. After that, the membrane was blocked with 3% nonfat dry milk (NFM) (Cat. # 170-6404, Bio-Rad) in Tris-buffered saline (TBS) (50 mM Tris, 150 mM NaCl, pH 7.5) containing 0.05% (wt/vol) Tween 20 (TBST) at room temperature. The membranes were then incubated overnight at 4 °C with the following primary antibodies diluted in 1% TBST (see Table 1 for antibodies used and dilutions). The membranes were washed three times in TBST for 5 min each. They were incubated at room temperature for 1 h with horseradish peroxidase-conjugated rabbit anti-mouse or anti-rabbit IgG secondary antibody (Sigma-Aldrich, anti-mouse Cat. # A9044, anti-rabbit Cat. # A0545) diluted 1:5000 in 1% TBST. Blots were subsequently washed three times with 1× TBST for 5 min each and developed using a commercial chemiluminescent reagent (Clarity, Bio-Rad-170-5061) and imaged using the ChemiDoc MP (Bio-Rad)^30^. All incubation steps carried out at 4 °C or RT were done with gentle shaking. The quantification of blots was done by using Image Lab 5.0. Uncropped and unprocessed scans of the blots are supplied as Supplementary Figs. 4–6.

**Table 1:** List of primary antibodies used with corresponding dilution and antibodies manufacturer’s catalogue number.

Primary antibodies	Dilution	Source	Identifier
sEH	1:1000	Custom made in-house	In-house^83^
mEH	1:1000	Custom made in-house	In-house^84^
GSTA1/2	1:2000	Santa Cruz	Cat # sc-398714

### Analysis of metabolomics data

The mass spectra of the oxylipins were processed via the MRM model in Analyst v.1.6.1 Software on the 4000 QTRAP mass spectrometer system (SCIEX, Redwood City, CA). Lipid metabolite identification was carried out using an in-house and public metabolite database. Oxygenated lipid species were quantified by referring their peak areas to those of the internal standards, and the calculated oxylipins concentration was normalized against their calibration curve with standards. The metabolic multivariate data analysis was implemented using an integrated web-based platform MetaboAnalyst version 6.0 (http://www.metaboanalyst.ca/) to analyze the distinct clustering among the control and prometryn groups. The lipidomics data were log-transformed and scaled using auto scaling (mean-centered and divided by the standard deviation of each variable) before the analysis. Oxylipins were represented on a heat map using Euclidean distance and Ward's clustering. Sparse partial linear square discriminant analysis (sPLS-DA) of LC–MS/MS lipidomics data was performed to determine the differences between control and prometryn treated groups. Volcano plot analysis was performed to show increased and decreased oxylipins based on the p-value and fold change. Missing values were imputed to 1/5 of the minimum sample value of each variable. A value of p < 0.05 was considered statistically significant.

### Statistical analysis

Unless otherwise stated, all data are reported as mean ± standard error of the mean (SEM). Statistical significance was determined using the Student’s *t*-test. Graphs data analysis was carried out using GraphPad Prism 9.3.1 software (GraphPad Software, San Diego, CA). A value of p < 0.05 was considered statistically significant.

## Results

### Oxylipins profiling in control and prometryn treated mice liver

**Figure 1:**
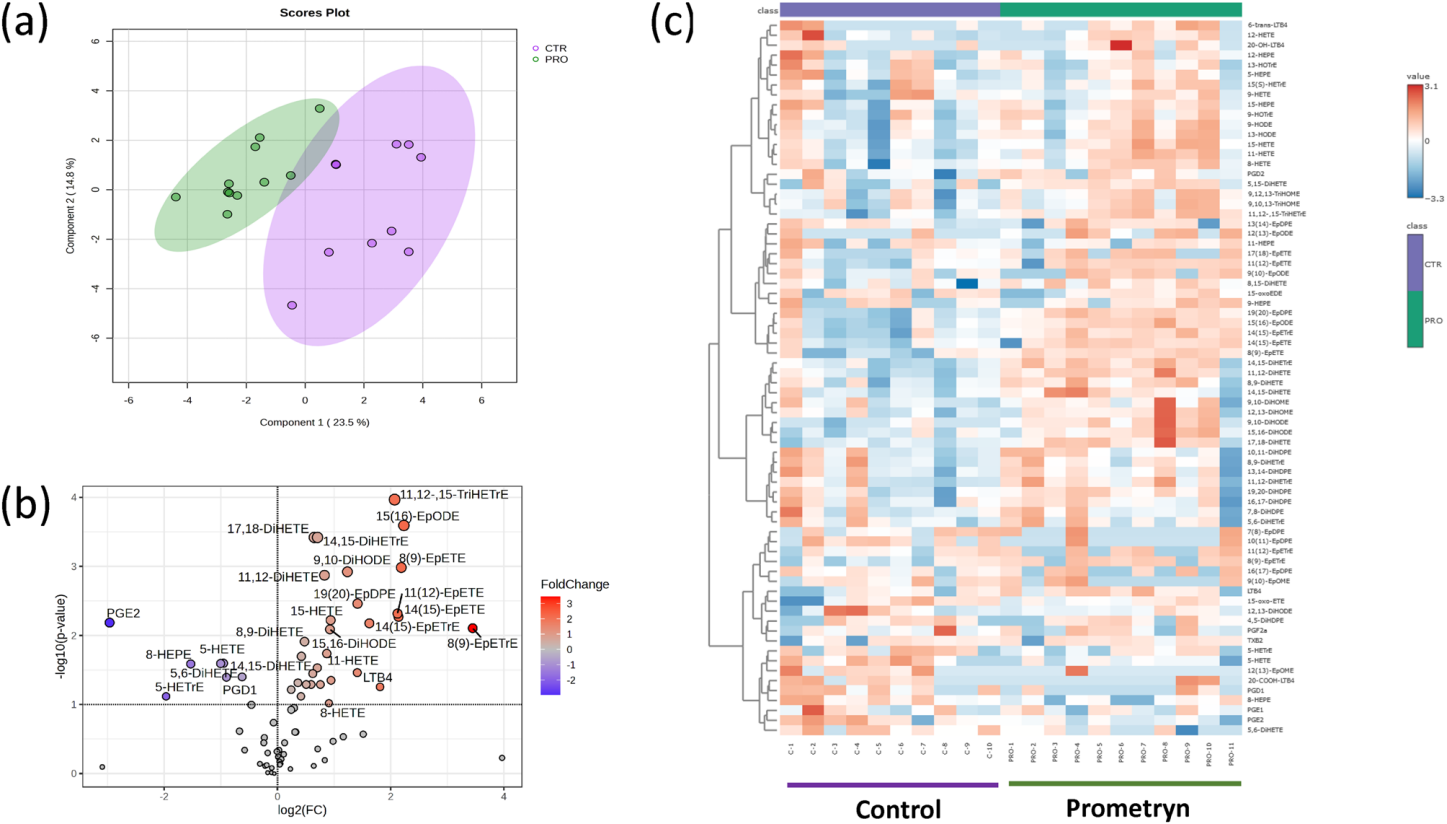
Short-term prometryn treatment altered oxylipin profiles in murine liver. (**a**) Sparse partial least squares-discriminant analysis (sPLS-DA) score plots of male mice livers treated with vehicle control and prometryn. (**b**) Volcano plot showing the significantly changed oxylipins profiles in mice liver after prometryn treatment. (**c**) Heat map showing AA, EPA, DHA, LA, and ALA metabolites profile shifted in vehicle control and prometryn treated groups in male mice liver. The relative intensities of variables in control and prometryn treated livers were shown by color bars. The color scale from − 3 to 3 represents the *z*-score. A positive *z*-score (increase in brown color intensity) reflects increased metabolite concentration and a negative *z*-score (increase in blue color intensity) reflects decreased concentration. Purple and green colored boxes on top of the heatmap represent the two groups (purple = control group and green = prometryn group), with their position showing how they cluster together, as determined by hierarchical clustering using Ward's algorithm.

To understand the effects and roles of oxylipin metabolites in prometryn treated mice, LC–MS/MS-based lipidomics was utilized to compare the profiles of oxylipin metabolites in the liver tissues of control and prometryn treated mice. Sparse partial linear square discriminant analysis (sPLS-DA) revealed the separation of samples and distinct oxylipin profiles in the livers of the control and prometryn groups (Fig. 1a). Specifically, a large number of oxylipins were significantly different between the control and prometryn groups (Fig. 1b). Heat map analysis revealed prometryn altered hepatic oxylipin levels (Fig. 1c). Notably, the most abundant oxylipins changes in prometryn treated mice liver were related to the CYP pathway followed by LOX-derived oxylipin metabolites. In contrast, minimal changes in COX-derived oxylipin metabolites were observed (Table 2). Therefore, the present study focused mainly on the CYP- and LOX-derived oxylipins.

**Table 2:** Levels of oxylipins that were significantly altered by prometryn in mice liver tissue.

Oxylipin	Control (n = 10)	Prometryn (n = 11)	p-value
Epoxides			
14,15-EpETrE	4.54 ± 5.58	14.02 ± 7.84	< 0.001
15(16)-EpODE	27.62 ± 32.63	129.83 ± 99.83	< 0.001
8(9)-EpETE	6.41 ± 9.76	29.31 ± 15.63	< 0.0005
11(12)-EpETE	2.74 ± 3.58	11.93 ± 5.86	< 0.0005
14(15)-EpETE	1.83 ± 2.32	8.06 ± 4.38	< 0.0005
19(20)-EpDPE	17.83 ± 24.40	47.51 ± 18.22	< 0.001
Hydroxy fatty acids			
5-HETrE	10.13 ± 7.89	2.82 ± 2.04	< 0.05
9-HODE	74.21 ± 32.64	114.32 ± 45.20	< 0.05
13-HODE	76.17 ± 30.70	115.13 ± 48.27	< 0.05
5-HETE	1.26 ± 0.68	0.65 ± 0.23	< 0.05
11-HETE	22.77 ± 10.49	41.65 ± 17.89	< 0.001
15-HETE	21.07 ± 9.46	40.43 ± 19.75	< 0.05
8-HEPE	2.46 ± 2.03	0.91 ± 0.68	< 0.05
Diols			
9,10-DiHODE	0.23 ± 0.08	0.52 ± 0.32	< 0.05
15,16-DiHODE	1.85 ± 0.68	3.55 ± 1.95	< 0.05
14,15-DiHETrE	16.82 ± 3.80	27.49 ± 6.18	< 0.0005
8,9-DiHETE	3.032 ± 0.94	4.22 ± 1.13	< 0.05
8,15-DiHETE	23.33 ± 12.89	38.02 ± 14.79	< 0.05
11,12-DiHETE	5.50 ± 1.45	9.82 ± 3.10	< 0.001
14,15-DiHETE	15.46 ± 3.73	20.75 ± 6.92	< 0.05
17,18-DiHETE	28.29 ± 3.87	44.37 ± 14.28	< 0.001
Trihydroxy fatty acids			
9,12,13-TriHOME	27.29 ± 18.61	52.60 ± 28.84	< 0.05
11,12,15-TriHETrE	67.82 ± 40.46	284.72 ± 159.03	< 0.001

Results are in pmol/g, mean ± standard deviation (mean ± SD). Significance level for the comparison between control and prometryn treated groups using the procedures described in the methods section. The metabolites are arranged in each section by chain length, then double bond so that LA, ALA, AA, EPA, and DHA metabolites (if present) are separated.

### Effect of prometryn on arachidonic acid-derived CYP oxylipins

**Figure 2:**
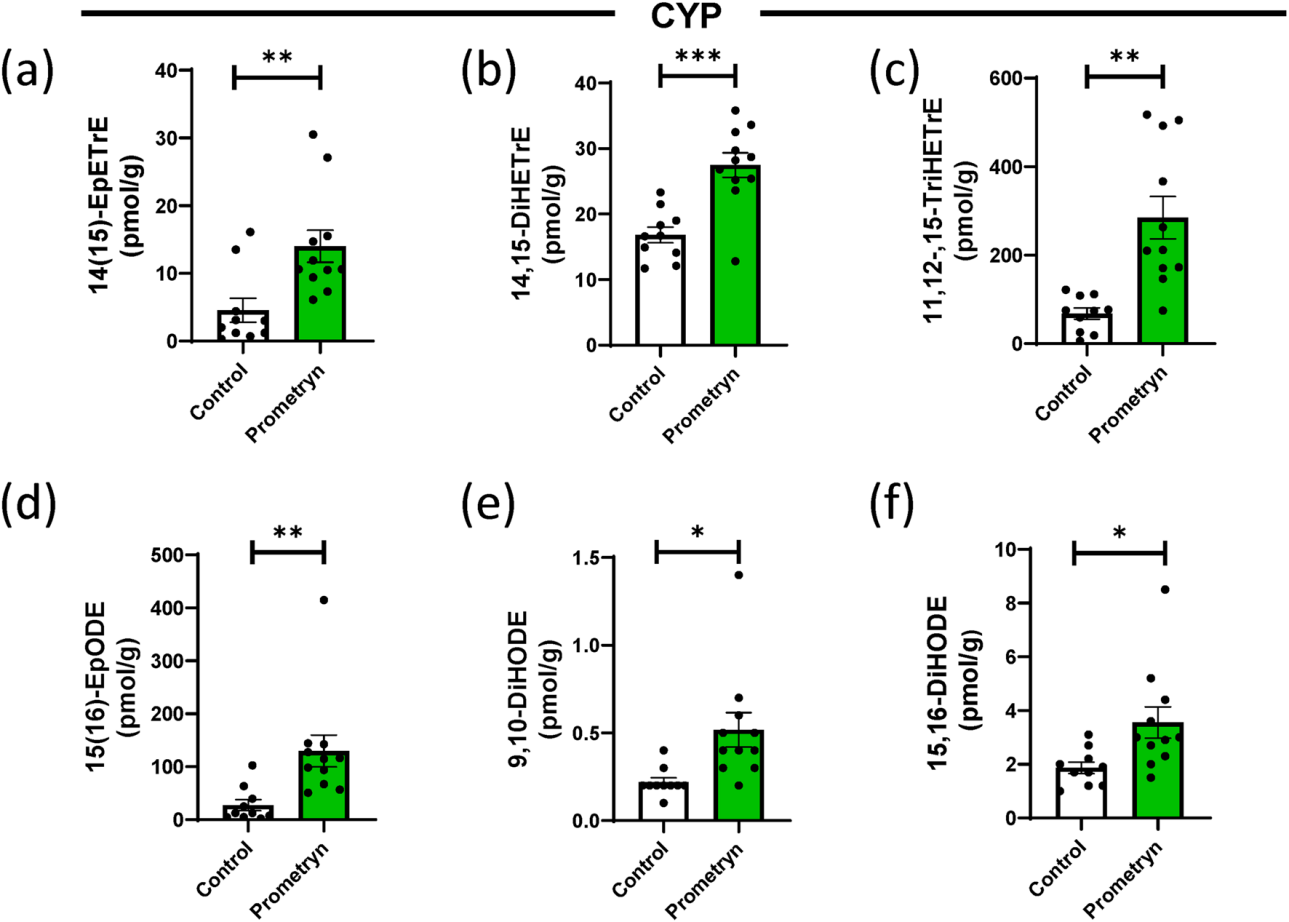
Prometryn increased the levels of CYP-derived oxylipins through the Arachidonic Acid (AA) pathway and α-linolenic acid (ALA) pathway. Plots for individual lipids showing that prometryn treatment increased the concentrations of CYP-derived oxylipins through the AA pathway. The concentrations of (**a**) 14(15)-EpETrE, (**b**) 14,15-DiHETrE, and (**c**) 11,12,15-TriHETrE were significantly increased in the liver of male mice exposed to prometryn for 7 days. Plots for individual lipids showing that prometryn treatment increased the concentrations of CYP-derived oxylipins through the ALA pathway. The concentrations of (**d**) 15(16)-EpODE, (**e**) 9,10-DiHODE, and (**f**) 15,16-DiHODE were significantly increased in prometryn treated mice liver when compared to their relative control liver. Bars represent the mean ± SEM; n = 10 to 11 mice per group. **p < 0.001, ***p < 0.0005.

CYPs utilize arachidonic acid (AA) to form oxylipins via epoxidation and hydroxylation activities, to yield epoxyeicosatrienoic acids (EpETrEs) and hydroxy-eicosatetraenoic acid (HETEs) respectively^21^. In this study, we found a significant increased level of 14,15-EpETrE, a labile vasodilatory eicosanoid^33^, in livers of mice treated with prometryn relative to the vehicle control treated mice livers (Fig. 2a, Table 2). The hepatic concentration of 14,15-EpETrE in control mice was 4.55 ± 1.76 pmol/g (mean ± SEM) while it was 14.01 ± 2.36 pmol/g in prometryn treated mice (p = 0.0048). We also found that 14,15-dihydroxy-eicosatrienoic acid (14,15-DiHETrE) which are fatty acid diols metabolized by sEH from 14(15)-EpETrE in the CYP-dependent AA pathway was significantly increased in prometryn treated male liver in contrast to the control male liver (Fig. 2b). The hepatic concentration of 14,15-DiHETrE in control mice was 16.82 ± 1.20 pmol/g while it was 27.48 ± 1.86 pmol/g in prometryn treated mice (p < 0.001). Prometryn treatment in mice also increased the hepatic concentration of 11,12,15-trihydroxyeicosatrienoic acid (11,12,15-TriHETrE) (Fig. 2c).

### Effect of prometryn on α-linoleic acid (ALA) derived CYP-oxylipins

CYP metabolizes ALA to produce epoxyoctadecadienoic acid (EpODE) which are subsequently hydrolyzed by sEH to yield dihydroxy-octadecadienoic acid (DiHODE). The lipidomic analysis showed that the levels of 15(16)-EpODE were significantly increased in prometryn treated group when compared to their respective control (Fig. 2d). Furthermore, the levels of sEH-produced metabolites 9,10-DiHODE and 15,16-DiHODE were also increased in the liver of prometryn mice when compared to the control mice (Fig. 2e,f). The hepatic concentrations of 15(16)-EpODE in vehicle control treated mice versus prometryn treated mice were 27.61 ± 10.32 pmol/g versus 129.8 ± 30.10 pmol/g (p = 0.0072), the concentrations of 9,10-DiHODE were 0.22 ± 0.02 pmol/g versus 0.51 ± 0.09 pmol/g (p < 0.05), and the concentrations of 15,16-DiHODE were 1.87 ± 0.21 pmol/g versus 3.55 ± 0.58 pmol/g (p < 0.05) (Table 2). Taken together, our data showed that prometryn increased the level of major CYP-derived oxylipins in various pathways including ALA and it particularly acts via increasing sEH activity/expression which eventually leads to an increase in sEH produced fatty acid diols in mice liver.

### Effect of prometryn on eicosapentaenoic acid-derived CYP oxylipins

**Figure 3:**
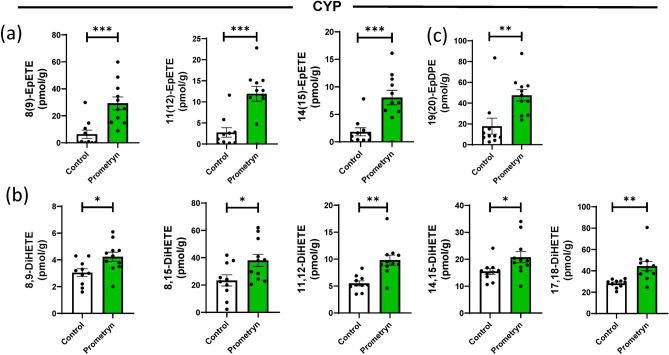
Prometryn increased the levels of CYP-derived oxylipins through the eicosapentaenoic acid (EPA) and docosahexaenoic acid (DHA) pathway. (**a**) Plots for individual lipids showing that prometryn treatment increased the concentrations of CYP-derived oxylipins through the EPA pathway. The concentrations of 8(9)-EpETE, 11(12)-EpETE, and 14(15)-EpETE were significantly increased in the liver of male mice exposed to prometryn for 7 days. (**b**) The concentrations of sEH-produced fatty acid diols via CYP metabolism of EPA pathway were significantly increased in the liver of mice exposed to prometryn. (**c**) Concentration of CYP-derived oxylipin through the DHA pathway in liver tissue of male mice exposed to prometryn was significantly increased. Bars represent the mean ± SEM; n = 10 to 11 mice per group. *p < 0.05, **p < 0.001, ***p < 0.0005.

Eicosapentaenoic acid (EPA) is an efficient alternative substrate to arachidonic acid (AA) metabolism by CYP^34^. Hepatic and renal CYP epoxygenases oxidize the ω-3 PUFA eicosapentaenoic acid (EPA) to epoxyeicosatetraenoic acids (EpETE) which are further converted into less-active dihydroxyeicosatetraenoic acids (DiHETE) by soluble epoxide hydrolase (sEH)^35,36^. The lipidomic analysis revealed that 8(9)-EpETE, 11(12)-EpETE, and 14(15)-EpETE were significantly increased in male livers treated with prometryn compared to their respective control (Fig. 3a). Interestingly, sEH-produced fatty acid diols appeared to be amongst the major metabolites contributing to the difference between the control and prometryn treated mice. 8,9-DiHETE, 8,15-DiHETE, 11,12-DiHETE, 14,15-DiHETE, and 17,18-DiHETE were significantly elevated in the liver of prometryn treated mice when compared to the vehicle control treated mice (Fig. 3b).

### Effect of prometryn on docosahexaenoic acid (DHA)-derived CYP oxylipins

CYP are also involved in the preferential epoxidation of ω-3 double bond of docosahexaenoic acid (DHA) to produce epoxydocosapentaenoic acids (EpDPE)^35^. EpDPE can be further converted to fatty acids diols by the action of sEH to generate less-active dihydroxy-docosapentaenoic acid (DiHDPE)^21^. Moreover, the most abundant DHA regioisomer synthesized by microsomes and by 15 human recombinant CYPs, including CYP2C8 and CYP2J2, is 19(20)-EpDPE^35^. We found that the level of 19(20)-EpDPE was significantly increased in prometryn treated male livers (Fig. 3c). The hepatic concentrations of 19(20)-EpDPE in vehicle control treated mice versus prometryn treated mice were 17.82 ± 7.71 pmol/g versus 47.50 ± 5.49 pmol/g (p = 0.0062).

### Prometryn altered the levels of lipoxygenase derived oxylipins in mice liver

**Figure 4:**
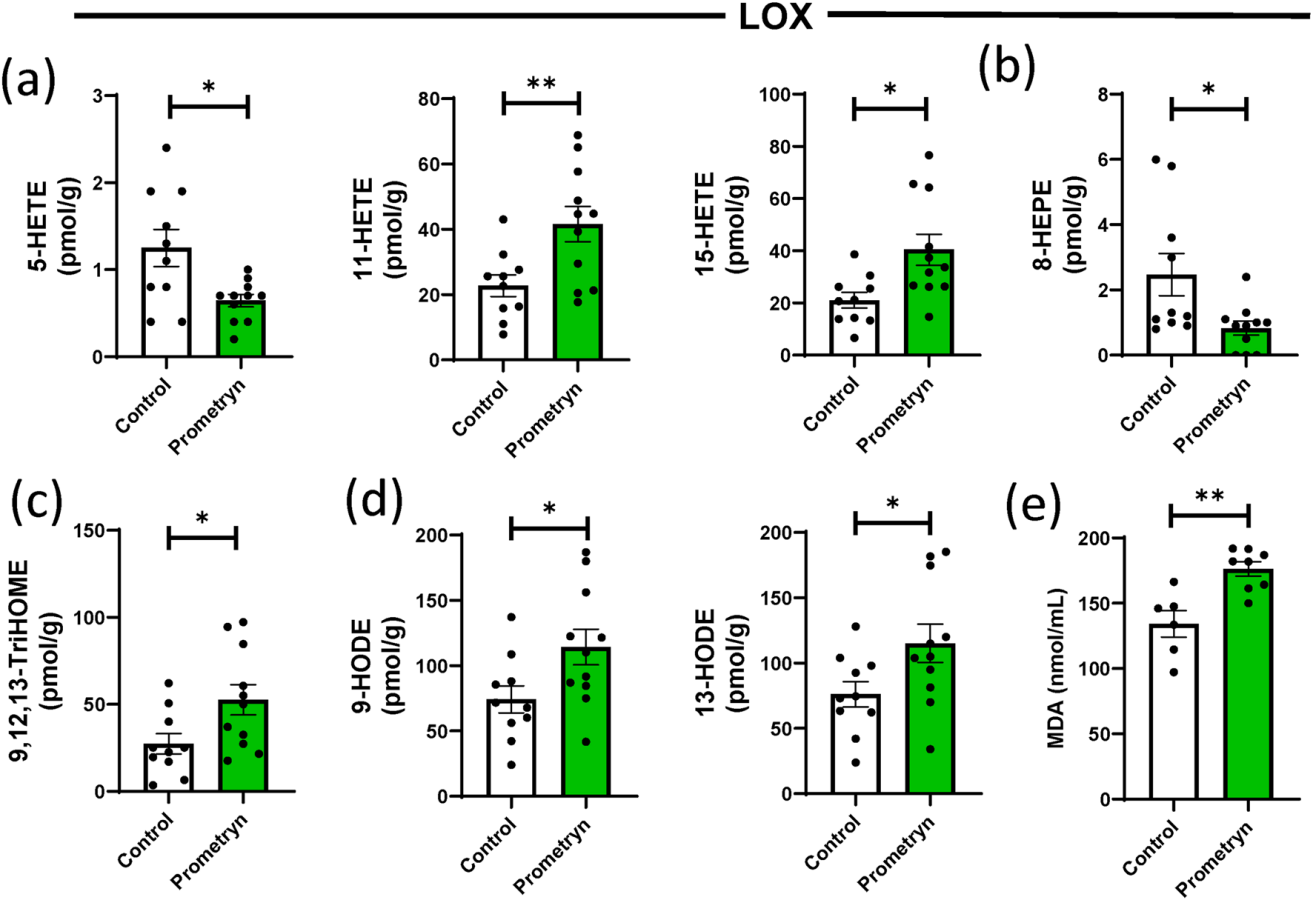
Prometryn altered the levels of lipoxygenase (LOX) pathway and increased lipid peroxidation in mice liver. (**a**) Plots for individual lipids showing that prometryn treatment increased the concentrations of LOX-derived oxylipins through the AA pathway. (**b**) Plots for individual lipids showing that prometryn treatment increased the concentrations of LOX-derived oxylipins through the EPA pathway. (**c**) Plots for individual lipids showing that prometryn treatment increased the concentrations of LOX-derived oxylipins through the LA pathway. (**d**) The concentrations of 9-HODE and 13-HODE, which are excellent biomarkers for lipid peroxidation, significantly increased in prometryn treated male mice liver. (**e**) Lipid peroxidation (measured by malondialdehyde reaction with thiobarbituric acid) was significantly increased in prometryn treated male mice liver when compared to vehicle control-treated male mice liver. Bars represent the mean ± SEM; n = 10 to 11 mice per group. *p < 0.05, **p < 0.001.

Lipoxygenases (LOX) incorporate two oxygen atoms into their substrate in order to form hydroperoxyl derivatives of fatty acids. LOX enzymes are typically classified based on their formation of the resulting hydroxy derivatives such as 5-LOX, 8-LOX, 11-LOX, 12-LOX, and 15-LOX^3^. After 7 days of prometryn treatment in mice, our unbiased lipidomics analysis revealed that multiple LOX-derived oxylipins were altered in the liver of the treated group. LOX utilizes AA as a substrate to form hydroperoxyeicosatetraenoic acids (HpETEs) that ends up as oxylipins such as 5-HETE, 15-HETE, and 11-HETE. Surprisingly, we found that prometryn treatment significantly increased the levels of 11-HETE and 15-HETE while it decreased the level of 5-HETE in the male livers when compared to their relative control (Fig. 4a). The metabolism of EPA by 8-LOX produces 8-hydroperoxyeicosapentaenoic acid (8-HpEPE) which is a precursor of 8-hydroxyeicosapentaenoic acid (8-HEPE). The level of 8-HEPE was significantly decreased in mice livers treated with prometryn compared to the control groups (Fig. 4b). 15-LOX can convert linoleic acid to 13-hydroperoxyoctadecadienoic acid (13-HpODE), which acts as a precursor to form trihydroxyoctadecenoic acids (TriHOMEs)^37^. We found that the level of 9,12,13-TriHOME was significantly elevated in prometryn treated mice livers than the vehicle control treated mice livers (Fig. 4c). When LOX oxidizes LA, they can also form 9-HpODE and 13-HpODE which acts as precursor to yield 9-hydroxyoctadecadienoic acid (9-HODE) and 13-hydroxyoctadecadienoic acid (13-HODE), respectively. 9-HODE and 13-HODE are recognized as biomarkers of lipid peroxidation, a hallmark indicator of oxidative stress^7,38,39^. Prometryn group had a significantly increased levels of 9-HODE and 13-HODE when compared to control group (Fig. 4d). There was also a significant increase in the level of lipid peroxidation byproduct malondialdehyde (MDA) in the prometryn treated mice liver when compared to the control liver (Fig. 4e). Leukotriene B4 (LTB4), metabolized from 5-HpETEs via 5-LOX pathway, was uncovered to be significantly higher in prometryn group (Supplementary Fig. 1). Together, the data showed that prometryn treatment altered the hepatic concentrations of LOX-derived oxylipin notably the increase in 9,12,13-TriHOME, 9-HODE and 13-HODE. The data also showed that the observed changes in these oxylipins were most likely due to increased lipid peroxidation. We postulate from the data that prometryn could be hepatotoxic via the induction of oxidative damage in mice liver by increasing the oxidation of LOX-derived oxylipins.

### Prometryn increased antioxidant defense system protein in mice liver and elevated oxidative stress in HepG2 liver cells

**Figure 5:**
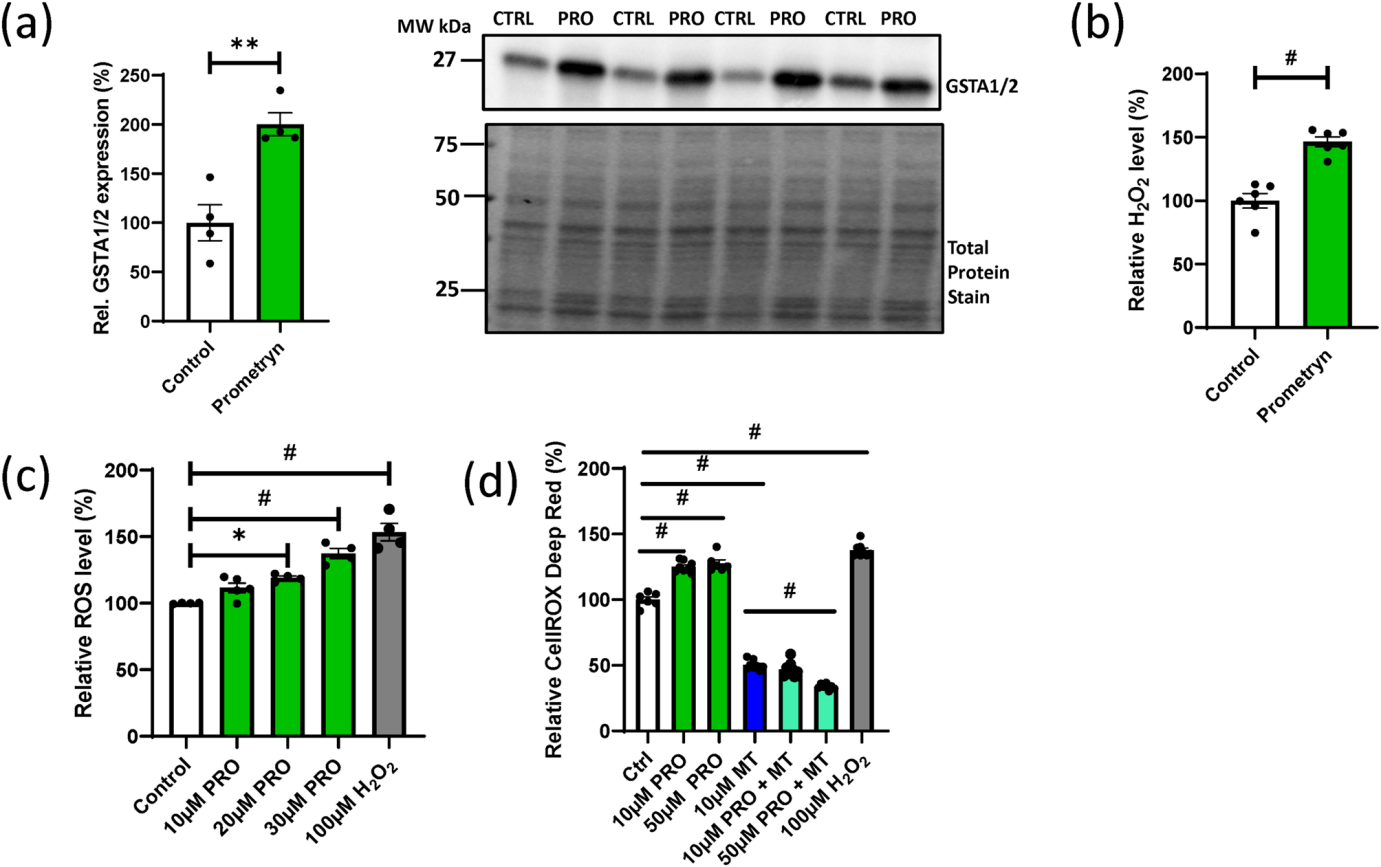
Short-term prometryn treatment induced oxidative-stress in mice liver. (**a**) Prometryn treatment significantly increased the protein expression of glutathione S-transferase alpha 1/2 (GSTA1/2) in mouse liver. Band signal intensities were analyzed by Image Lab^®^ Software Version 12 and normalized with intensities obtained from loading control after staining with commercial Ponceau S for total protein. (**b**) Relative hydrogen peroxide (H_2_O_2_) levels of control and prometryn treated liver. (**c**) ROS formation was detected in HepG2 liver cells stained with a cell permeable fluorescent probe H_2_DCFDA and treated with 10–30 μM prometryn. H_2_O_2_ (200 μM) was used as a positive oxidative stress control. ROS levels were detected by fluorescence spectroscopy at an Ex-502 nm and Em-523 nm. (**d**) Elevated oxidative stress in HepG2 liver cells treated with prometryn was confirmed by staining with CellROX Deep Red. Meanwhile, 1 h pretreatment with mitochondrial-targeted antioxidant MitoTEMPO significantly decreased the oxidative stress. Shown are spectrofluorometric data expressing the fluorescence intensity of MitoSOX after treatment with prometryn and/or MitoTEMPO. *PRO* Prometryn, *MT* MitoTEMPO, *MT + P* MitoTEMPO + Prometryn. Bars represent the mean ± SEM; n = 4–8 mice per group. *p < 0.05, **p < 0.001, # = p**** < 0.0001.

Given the findings that prometryn increased oxylipin concentrations associated with oxidative stress in mice liver, the protein expression of an antioxidant enzyme glutathione S-transferase alpha 1/2 (GSTA1/2) was investigated via immunoblotting. The expression level of GSTA1/2 was significantly upregulated in prometryn treated mice liver in comparison to vehicle-treated mice liver (Fig. 5a). Subsequently, hydrogen peroxide (H_2_O_2_) levels were measured in mice liver since we postulated that the increase in GSTA1/2 expression could be due to its role in detoxifying xenobiotics that cause oxidative damage. In prometryn treated mice liver, H_2_O_2_ was significantly increased than in control treated mice liver (Fig. 5b). H_2_O_2_ functions both as intracellular messenger and as a source of oxidative stress. H_2_O_2_ overproduction in intra- or extracellular spaces has been established to increase ROS level, eventually resulting in cell and tissue injury^7^. Furthermore, we investigated whether prometryn affects ROS levels in vitro by using HepG2 liver carcinoma cells. HepG2 cells were stained with the fluorescent probe H_2_DCFDA and CellROX Deep Red, and the changes in intracellular ROS levels were quantitatively detected via fluorescence intensity. Prometryn treatment resulted in an elevated ROS generation in a concentration-dependent manner. 100 μM H_2_O_2_ was used as a positive control for ROS formation (Fig. 5c,d). We observed that mitochondrial superoxide scavenger MitoTEMPO (10 μM) prevented an increase in ROS level in the presence of prometryn (Fig. 5d). Our in vitro data showed that prometryn increased intracellular ROS level in HepG2 cells even at low concentrations of 10–30 μM while in vivo data also showed an increase in GSTA1/2 protein expression and H_2_O_2_ level in prometryn treated mice liver, corroborating our postulation that prometryn elicits hepatotoxicity in mice by inducing oxidative stress.

### Prometryn increased mEH and sEH expression and activities in mice liver

**Figure 6:**
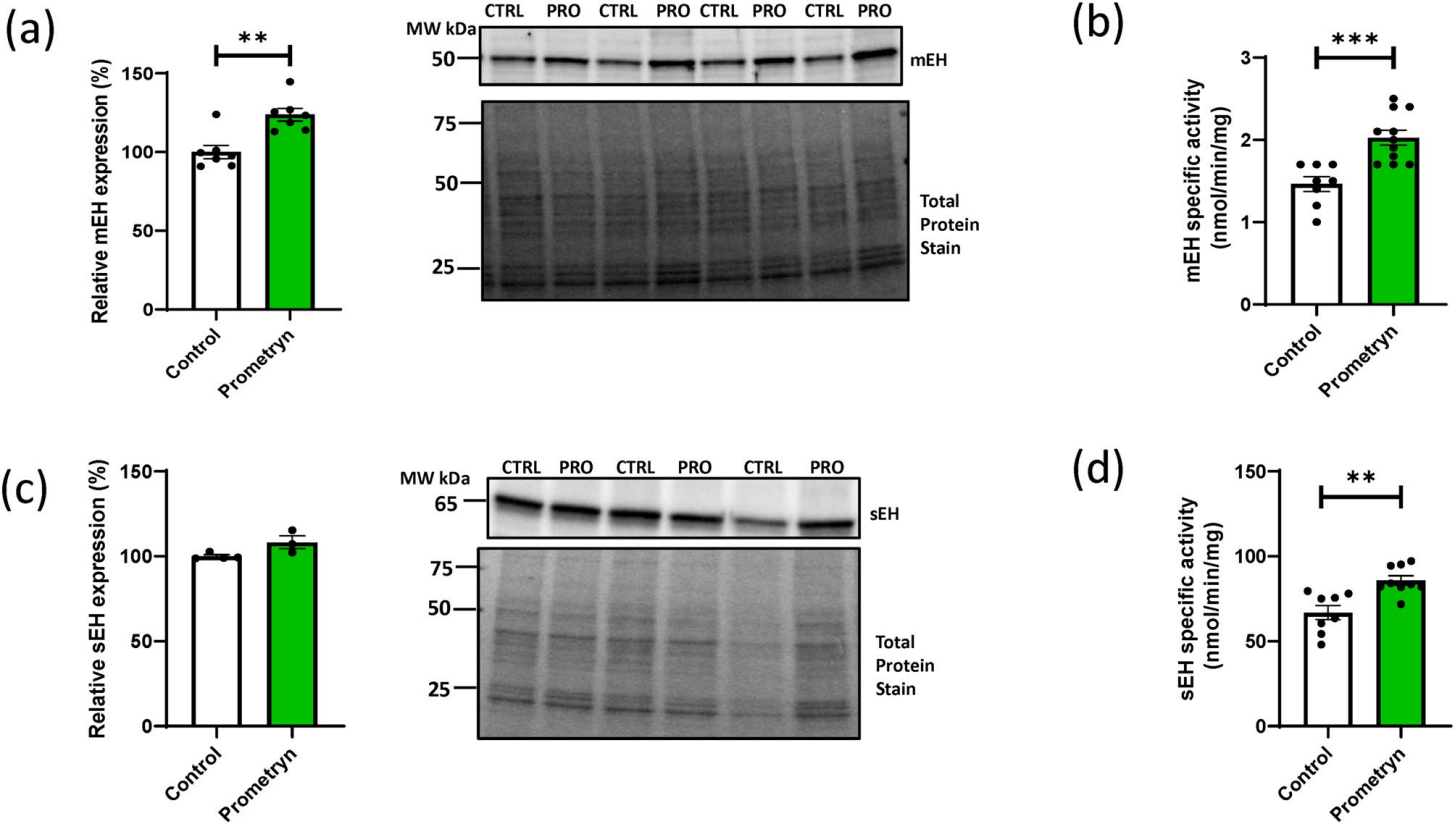
Prometryn treatment increased the enzymatic activities of microsomal and soluble epoxide hydrolase (mEH and sEH) in mice liver. (**a**) Relative expression of mEH in livers from male mice treated with vehicle control and prometryn every 48 h for 7 days as determined by immunoblotting (n = 8). Band signal intensities were analyzed by Image Lab^®^ Software Version 12 and normalized with intensities obtained from loading control after staining with commercial Ponceau S for total protein. (**b**) mEH enzymatic activity in control and prometryn treated male mouse liver (n = 8–11). (**c**) Relative expression of sEH in livers from male mice treated with vehicle control and prometryn every 48 h for 7 days as determined by immunoblotting (n = 8). Band signal intensities were analyzed by Image Lab^®^ Software Version 12 and normalized with intensities obtained from loading control after staining with commercial Ponceau S for total protein. (**d**) sEH enzymatic activity in control and prometryn treated male mouse liver (n = 8–11). Bars represent the mean ± SEM. **p < 0.001, ***p < 0.0005.

**Figure 7:**
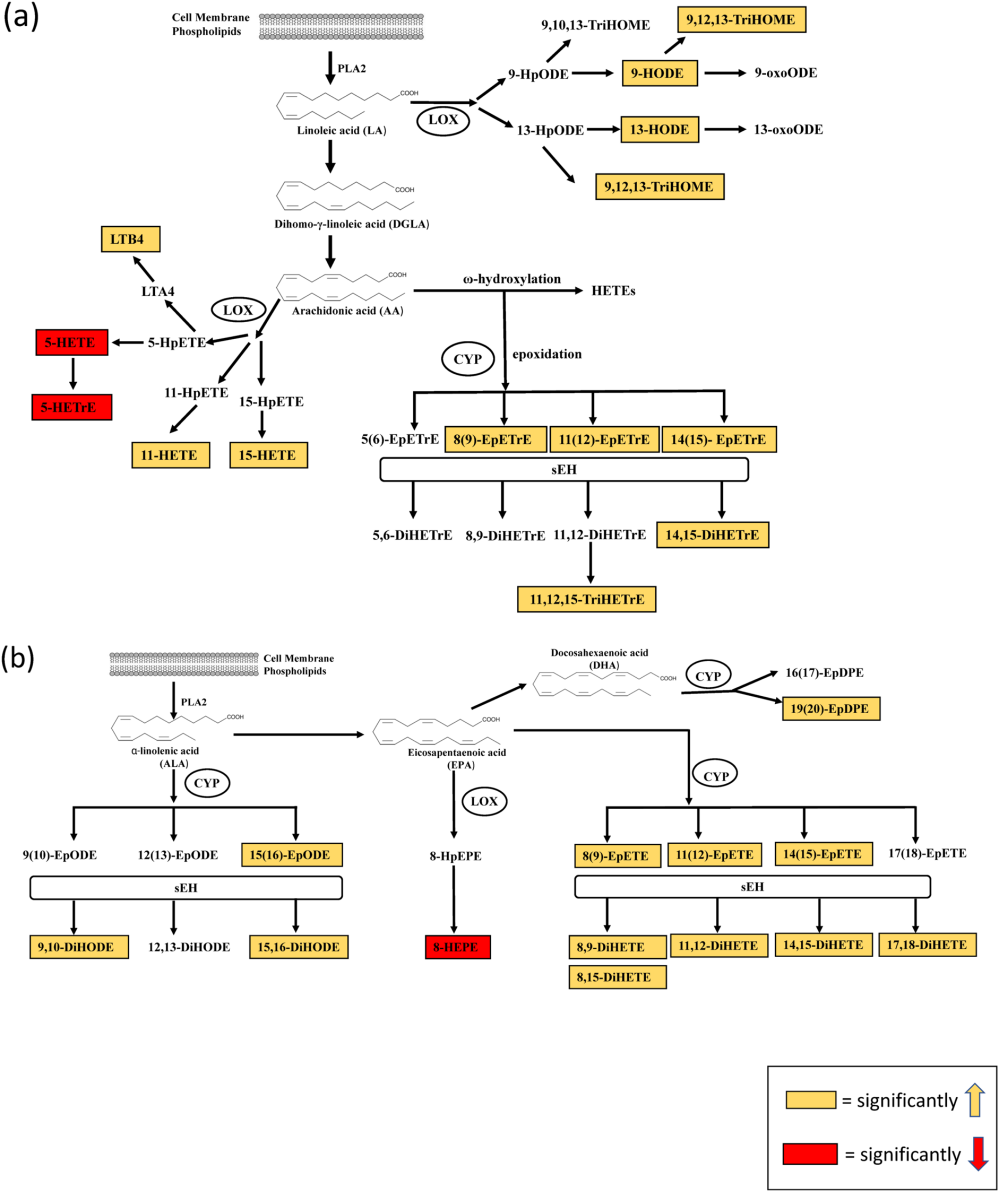
Pathway analysis. (**a**) Schematic diagram showing the activities of LOX and CYP450 enzymes that catalyze the formation of oxylipins species with different biological activities starting from omega-6 PUFAs precursors in mice liver treated with prometryn every 48 h for 7 days. Prometryn treated mice had marked alterations in the CYP450 pathway and minor alterations in the LOX pathways resulting in the increase of oxylipins (gold) and decrease of oxylipins (red). (**b**) Schematic diagram showing the activities of LOX and CYP450 enzymes that catalyze the formation of oxylipins species with different biological activities starting from omega-3 PUFAs precursors in mice liver treated with prometryn every 48 h for 7 days. Prometryn treated mice had marked alterations in the CYP450 pathway and minor alterations in the LOX pathways resulting in the increase of oxylipins (gold) and decrease of oxylipins (red).

Since prometryn increased the concentrations of sEH-produced fatty acid diols in mice liver from the lipidomics analysis, we investigated whether prometryn altered mEH and sEH expression and activities in mice liver. sEH is recognized as a promising target for the treatment of hypertension, inflammatory diseases, pain, diabetes, and stroke^40^. Notably, prometryn significantly increased both the protein expression level (Fig. 6a) and specific activity (Fig. 6b) of mEH in mice liver. Prometryn had no significant change on sEH protein expression level (Fig. 6c) but it significantly increased the specific activity of sEH in mice liver (Fig. 6d). The data showed that low prometryn treatment to mice significantly increased mEH and sEH activities in mice livers relative to their controls, thereby suggesting that the increased oxidative stress that was earlier observed might be due to the increase in sEH. This is the first report of mEH and sEH being upregulated by prometryn treatment in mice or any other organism model. Figure 7a,b shows a schematic diagram of the oxylipins that were altered by prometryn treatment in mice liver.

### Prometryn altered circulatory oxylipins in mice

**Figure 8:**
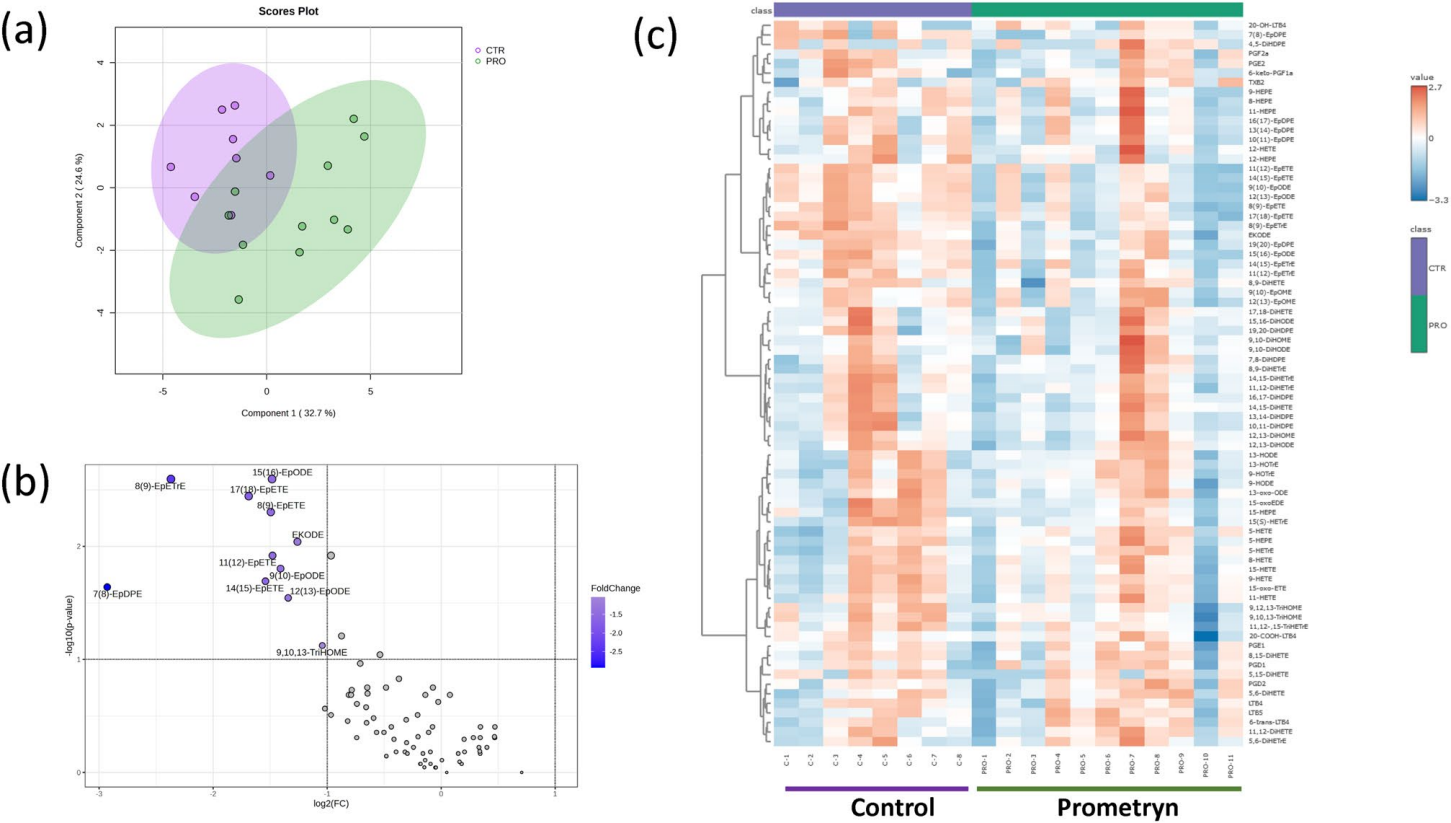
Oxylipin profiling in plasma from prometryn treated and control mice. (**a**) Sparse partial least squares-discriminant analysis (sPLS-DA) score plots of mice plasma treated with vehicle control and prometryn. (**b**) Volcano plot showing the fold change of significant oxylipins profile in vehicle control and prometryn treated groups in male mice plasma. (**c**) Heat map showing oxylipins profile shifted in vehicle control and prometryn treated groups in male mice plasma.

**Figure 9:**
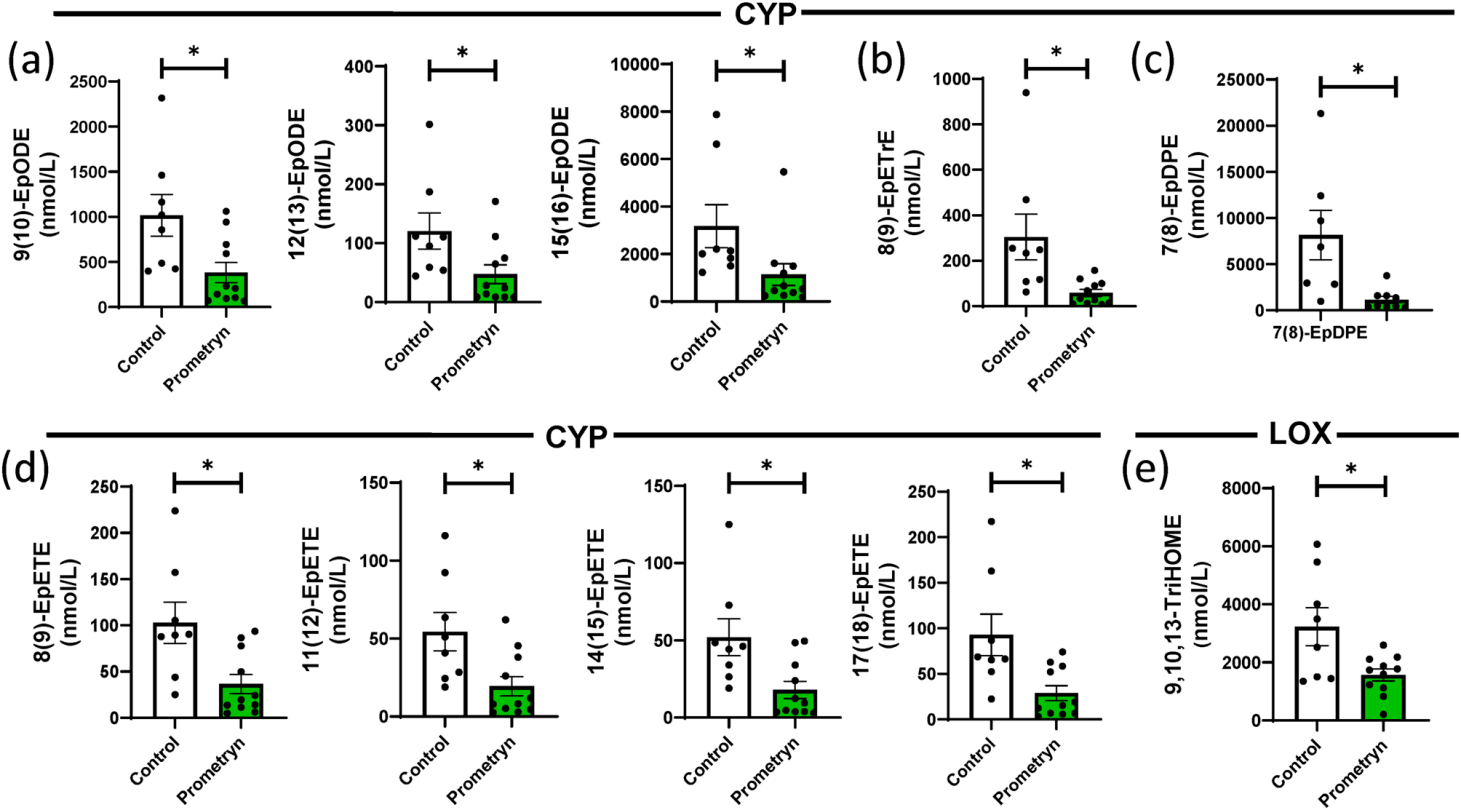
LC–MS/MS-based lipidomics showed that prometryn decreased circulatory (plasma) oxylipin levels in mice. (**a**) Plots for individual lipids showing that prometryn treatment significantly decreased the concentrations of CYP-derived oxylipins through the ALA pathway. (**b**) Plots for individual lipids showing that prometryn treatment significantly decreased the concentrations of CYP-derived oxylipins through the AA pathway. (**c**) Plots for individual lipids showing that prometryn treatment significantly decreased the concentrations of CYP-derived oxylipins through the DHA pathway. (**d**) Plots for individual lipids showing that prometryn treatment significantly decreased the concentrations of CYP-derived oxylipins through the EPA pathway. (**e**) A plot for 9,10,13-TriHOME showed that prometryn treatment significantly decreased the concentration of this LOX-derived oxylipin. Bars represent the mean ± SEM; n = 8 to 11 mice per group. *p < 0.05.

**Figure 10:**
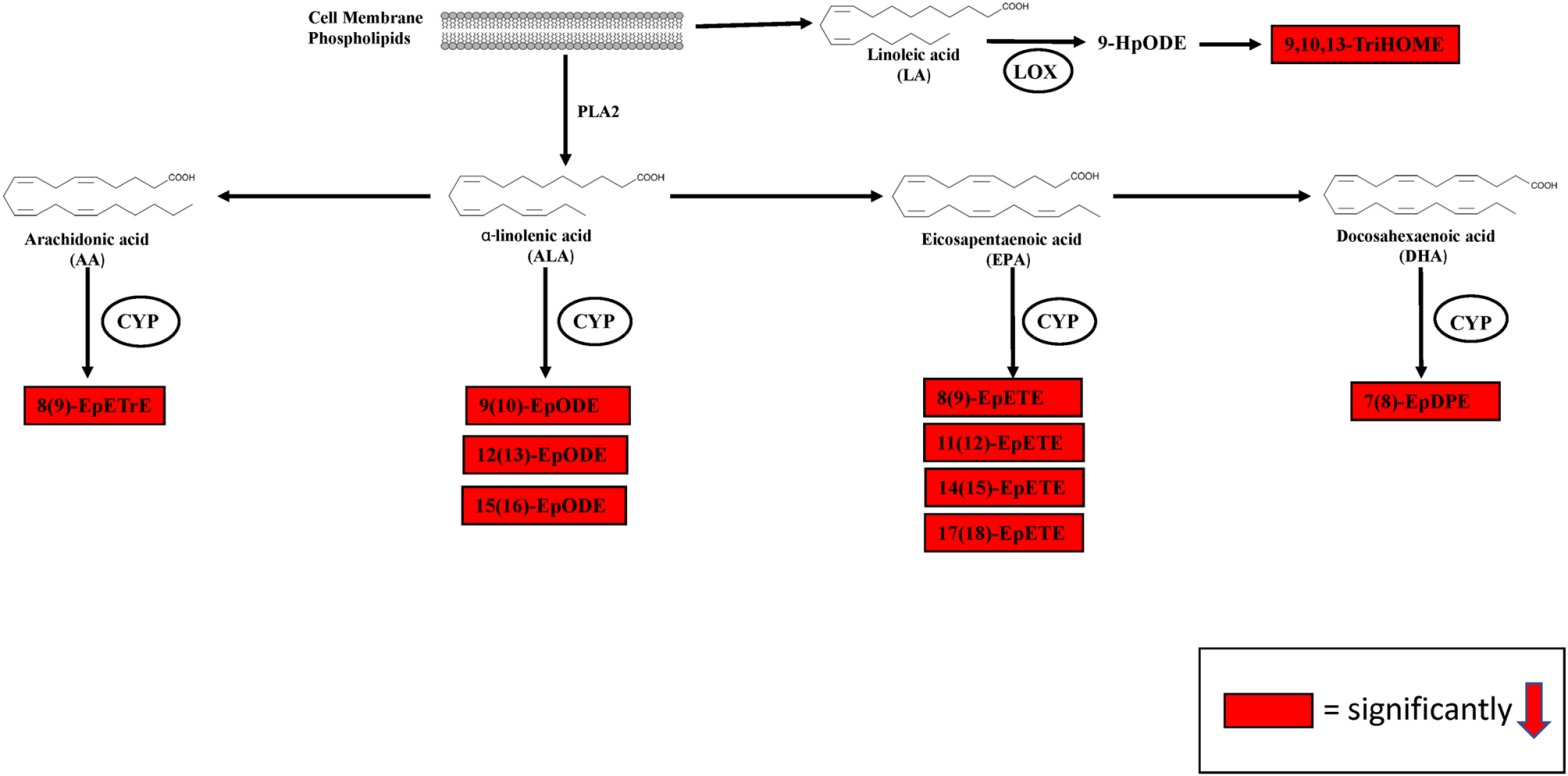
Pathway analysis (plasma). Schematic diagram showing oxylipins that were altered by prometryn treatment in mice circulatory (plasma). Red indicates that oxylipin significantly decreased.

The LC–MS/MS-based lipidomics was also used to analyze the oxylipin profile in the plasma of vehicle control and prometryn treated mice. sPLS-DA, volcano plot, and heatmap analyses showed a difference in the plasma oxylipin profiles between the control and prometryn groups (Fig. 8a–c). Among the detected metabolites, the plasma concentrations of a series of CYP-derived metabolites from ALA pathway, including 9(10)-EpODE, 12(13)-EpODE, 15(16)-EpODE were significantly decreased in prometryn treated mice plasma when compared to controls (Fig. 9a). 8(9)-EpETrE, a CYP-derived oxylipin from the AA pathway, was significantly decreased in prometryn treated mice plasma compared to controls (Fig. 9b). 7(8)-EpDPE, which is a CYP-derived oxylipin from the DHA pathway, was significantly decreased in prometryn treated mice plasma when compared to controls (Fig. 9c). Additionally, CYP-derived oxylipins from EPA pathway such as 8(9)-EpETE, 11(12)-EpETE, 14(15)-EpETE, and 17(18)-EpETE were also significantly decreased in prometryn treated mice plasma when compared to controls (Fig. 9d). Finally, the lipidomic analysis showed that prometryn significantly decreased the concentration of a LOX-derived oxylipin, 9,10,13-TriHOME in mice plasma (Fig. 9e, Table 3). Our data suggests that prometryn mainly targets the CYP enzymes in mice's circulatory system (Fig. 10).

**Table 3:** Levels of oxylipins that were significantly altered by prometryn in plasma of mice.

Oxylipin	Control (n = 8)	Prometryn (n = 11)	p-value
Epoxides			
8(9)-EpETrE	304.53 ± 285.84	58.90 ± 51.90	< 0.05
9(10)-EpODE	1016.81 ± 649.32	382.99 ± 371.97	< 0.05
12(13)-EpODE	120.59 ± 86.09	47.57 ± 53.31	< 0.05
15(16)-EpODE	3177.93 ± 2557.68	1136.46 ± 1521.93	< 0.05
8(9)-EpETE	102.88 ± 62.89	36.52 ± 34.23	< 0.05
11(12)-EpETE	54.44 ± 34.54	19.53 ± 20.46	< 0.05
14(15)-EpETE	52.05 ± 33.63	17.90 ± 18.31	< 0.05
17(18)-EpETE	92.70 ± 64.40	28.77 ± 27.10	< 0.05
7(8)-EpDPE	8146.75 ± 7102.16	1132.57 ± 1106.06	< 0.05
Trihydroxy fatty acids			
9,10,13-TriHOME	3233.00 ± 1848.24	1569.55 ± 677.68	< 0.05

Results are in nmol/L, mean ± standard deviation (mean ± SD). Significance level for the comparison between control and prometryn treated groups using the procedures described in the methods section. The metabolites are arranged in each section by chain length, then double bond so that LA, ALA, AA, EPA, and DHA metabolites (if present) are separated.

### Prometryn altered proteasome activity in mice liver

We previously found that mice given a 15-HETE diet showed altered expression of proteasome and the immunoproteasome catalytic subunits, suggesting that the activities of proteasomes and immunoproteasomes may be affected by increases in 15-HETE levels^41^. Since 15-HETE levels were increased in livers from prometryn treated mice, we investigated proteasome and immunoproteasome activities in treated and control mouse livers. Prometryn increased proteasome β1 and β2 activities and decreased β5 activity in mice livers (Supplementary Fig. 2a). Immunoproteasome activities β1i and β5i were not significantly affected by prometryn treatment (Supplementary Fig. 2b).

## Discussion

The present study examined the liver and plasma lipidome of mice exposed to vehicle control and mice given 185 mg/kg prometryn every 48 h for seven days using LC–MS/MS lipidomics approach. The concentration of prometryn used in this animal study was similar to the lowest concentrations used in previous studies^22–24^. The dose of 185 mg/kg of prometryn used in this study corresponds to 1/20 of the LD50 for the mouse. To our knowledge, this is the first study profiling oxylipin metabolites of mice treated with prometryn or any triazine herbicides. This study specifically aimed to measure the liver oxylipin changes due to the effect of prometryn. The plasma oxylipins were also analysed to understand the similarities and variations between the liver and the plasma. Plasma was also chosen because it circulates throughout the body, carrying information and substances to various tissues and organs. As a result, studying plasma provides a systemic perspective, offering insights into the overall health and functioning of the body rather than focusing on specific tissues or organs alone. We chose oxylipins over other lipids for the lipid studies based on their important roles and potent oxylipin bioactivities. There are very few studies of the biological functions of oxylipins due to their lower abundance and difficulty in analyzing them.

Seventy-three and seventy-six oxylipin metabolites were detected and quantified in liver tissues and plasma of mice, respectively (Supplementary Tables 1 and 2; some oxylipin metabolites were below the limit of detection or quantitation of the LC–MS/MS method). The Limit Of Quantification (LOQ) for the oxylipins detected are shown in Supplementary Table 3. Exposure to prometryn significantly changed the concentration of twenty-two oxylipins in mice liver and the concentration of ten oxylipins in mice plasma. The oxylipins significantly changed in this study were derived from different precursors, including AA, EPA, DHA, and ALA, via CYP and LOX enzymes only. Little significant changes were found in oxylipins derived via COX enzymes.

To determine if the corn oil contains significant amounts of oxylipins, two aliquots of frozen corn oil used as the vehicle for these experiments were analyzed. The results suggest that 38 out of the 65 oxylipins detected had an average value of less than 0.1 nmol/L, and 59 out of the 65 had an average value of less than 1 nmol/L (Supplementary Table 4). The individual values from the liver and plasma samples are included in Supplementary Table 5. To determine the reproducibility of oxylipin detection from liver samples, experiments were done by extracting 100 mg of liver tissue in triplicate and running them through the same protocol. The relative standard deviation (RSD) data shows the data variations were well controlled despite any matrix effects (Supplementary Table 6).

There are 18 mammalian CYP families, some of which encode enzymes involved in eicosanoid metabolism, while other CYP families are predominantly involved in the detoxification process of xenobiotics and biosynthesis of other chemical mediators such as steroid hormones and other products of endogenous metabolism^21,42^. CYP2, CYP3, and CYP4 families contain far more genes than the other 15 families; these three families are also the ones that are dramatically larger in rodent genomes^42^. CYP enzymes form oxylipins via their epoxygenase and ω-hydroxylase activities^3^. CYP2C and CYP2J epoxygenases convert AA into EpETrE regioisomers (primarily 11,12- and 14,15-EpETrE), which are further converted into less-active dihydroxyeicosatrienoic acids (DiHETrE) by soluble epoxide hydrolase (sEH)^43^. EpETrEs are endogenous vasodilators in multiple vascular beds. The potency of 14,15-EpETrE to act as a vasodilator varies depending on the tissue and vessel size^44^. EpETrEs are synthesized by the vascular endothelium and released in response to vasoactive agonists^45^. Prometryn treatment in mice significantly increased the hepatic levels of 14,15-EpETrE, 14,15-DiHETrE, and 11,12,15-TriHETrE compared to controls. Luo et al. showed that the serum level of 14,15-EpETrE was increased in breast cancer patients and 14,15-EpETrE level in tumor tissue was higher than that of non-cancerous tissue. They also found that 14,15-EpETrE induced breast cancer cells chemotherapy resistance^46^. Another study also found a similar result where 14,15-EpETrE was elevated in breast cancer human tissue than in adjacent noncancerous human tissue^47^. Surprisingly, when chick embryos were exposed to an environmental toxin and carcinogen 2,3,7,8-tetrachlorodibenzop-dioxin (TCDD, dioxin), they found that it also increased the hepatic level of 14,15-EpETrE^48^. Additionally, a study showed that the hepatic level of 14,15-EpETrE was significantly increased in pair-fed and ethanol-fed mice expressing liver-specific sEH deletion, with these mice showing substantial increases in hepatic injury, inflammation, and steatosis^49^. Similar to our result, 14,15- DiHETrE was significantly elevated in a rat model with salt-sensitive hypertension^50^. The reduction in the production of DiHETrE through pharmacological sEH inhibition induced a reduction in blood pressure in models of hypertension, reduced vascular smooth muscle cell proliferation, and inhibited inflammatory pain processes^3^. Together, our data suggest that prometryn increased CYP, leading to a significant increase in 14,15-EpETrE. Sequentially, prometryn significantly elevated sEH activity, which led to an increase in the rapidly hydrolyzed sEH-produced oxylipin 14,15-DiHETrE. Therefore, targeting or inhibiting sEH might be a viable therapeutic target for prometryn induced liver injury. Inhibition of sEH has been shown to alleviate oxidative stress in carbon tetrachloride (CCl_4_)-induced cirrhosis in rats^51^; reduces portal pressure, liver fibrosis and attenuates hepatic endothelial dysfunction in cirrhotic rats^52^; and reduce oxidative stress, fibrosis, and electrical remodeling in thoracic aortic constriction mice model^53^.

Analogs of oxylipins from EPA, DHA, and ALA are also formed by CYP epoxygenase activity. It is interesting that the CYP-derived oxylipins that were significantly altered by prometryn in mice livers were as a result of epoxidation. Epoxy fatty acids (EpFA) are produced in response to platelet aggregation, vascular endothelial inflammation and can also function as vasodilators^21^. CYP-derived epoxy metabolites of DHA have been previously shown to possess anti-angiogenic, vasodilatory, and anticancer effects^54^. The levels of 8(9)-EpETE, 11(12)-EpETE, 14(15)-EpETE (derived from EPA), 19(20)-EpDPE (derived from DHA), and 15(16)-EpODE (derived from ALA) were significantly increased in prometryn treated male livers compared to controls. 19(20)-EpDPE (derived from DHA) was previously shown to suppress the formation of hepatic crown-like structure and nonalcoholic steatohepatitis fibrosis via G protein-coupled receptor 120^55^. The authors implicated the elevation of 19(20)-EpDPE in increasing eosinophils and monocyte-derived macrophages which play a role in liver regeneration in CCl_4_ liver-injury mice model and resolution of inflammation in acute peritonitis mice model^55^. This suggests that the increased hepatic level of 19(20)-EpDPE in prometryn treated mice might be partially or in part due to prometryn effects, which causes the recruitment of eosinophils to the injured liver. However, further studies are needed. Consistently, our results are largely in agreement with previous studies that found that ibuprofen treatment altered the levels of several EpFA oxylipins in male mice liver while also increasing the expression and enzymatic activities of sEH^21^. sEH is a regulatory enzyme that has C-terminal epoxide hydrolase and N-terminal lipid phosphatase activity. The epoxide hydrolase activity of sEH has a high affinity for epoxides of fatty acids, and it has also been associated with endothelial nitric oxide synthase (eNOS) dysfunction^56^. Inhibiting sEH maintains the level of endogenous bioactive EpFA and allows them to exert their generally beneficial effects^57^. In fact, Kim et al. showed that inhibition of the hydrolase activity of sEH enhanced levels of EET regioisomers and abolished tubulointerstitial fibrosis and inflammation in mice^58^. Remarkably, we found that prometryn increased the levels of sEH produced oxylipins in male mice. 8,9-DiHETE, 11,12-DiHETE, 14,15-DiHETE, and 17,18-DiHETE (derived from sEH hydrolysis of EpETE) as well as 9,10-DiHODE and 15,16-DiHODE (derived from sEH hydrolysis of EpODE) were significantly increased in prometryn treated male mice. Surprisingly, a study showed that 14,15-DiHETE and 17,18-DiHETE were both elevated in type 2 diabetic human subjects^1^. The higher expression levels and enzymatic activities of both sEH and mEH observed in the livers of prometryn treated mice may be crucial for the reduction of the elevated levels of EpETrEs. Nevertheless, sEH is the major enzyme that converts anti-inflammatory eicosanoids EpETrEs to DiHETrEs that are usually biologically inactive or proinflammatory in action^25^. Therefore, enhanced expression of sEH in liver tissues could lead to an increased inflammatory state, and some research studies have suggested that the sEH enzyme itself can be considered as an inflammatory marker^25^. Inhibition of sEH increases concentrations of the more vasodilatory EpETrEs by preventing their metabolism to the DiHETrEs that have less vasodilatory activity^59,60^.

The ubiquitin–proteasome pathway (UPS) is the major intracellular housekeeping system. The UPS degrades non-lysosomal, short-lived eukaryotic proteins, misfolded, as well as oxidized and damaged proteins. Decreases in proteasome activity has been associated with a buildup of misfolded proteins and the development of various diseases including cardiovascular diseases^61^. Recently, associations between oxylipins and proteasome expression has been reported. 20-HETE and high salt synergistically activate UPS via upregulation of one of the catalytic proteasome subunits^62^. Mice given a 15-HETE diet had altered expression of proteasome and the immunoproteasome catalytic subunits^41^. Since prometryn increased 15-HETE, proteasome and immunoproteasome activities were evaluated to determine if these activities were altered. Prometryn increased proteasome caspase like β1 and trypsin like β2 activities while chymotrypsin like β5 activity was decreased in mice livers. These results suggest that proteasome function is altered by prometryn and it is possible that these changes in proteasome activity may be partly caused by changes in oxylipin amounts.

Besides CYP-derived oxylipins, prometryn altered the oxylipin metabolites derived from LOX enzymes. LOX acts as a catalyst in the conversion of AA, LA, and EPA to labile hydroperoxy intermediates that are further synthesized to form hydroxyeicosatetraenoic acids (HETEs), leukotrienes, lipoxins, and hepoxillins that possess additional biological activities^21,63^. The LOX-derived oxylipins have been shown to be involved in various important pathological conditions and processes such as inflammation, allergic reactions, bronchoconstriction, and vasoconstriction, intracellular signaling, and cellular proliferation^64^. 5-HETE is reported to aid chemotaxis and promote neutrophil recruitment, the immune system's first line of defense^65^. In fact, 5-HETE was found to be the most chemotactic of the HETE family^66^. However, a significant decrease in the levels of 5-HETE was observed in prometryn treated mice liver, suggesting that prometryn could affect the immune system by reducing the recruitment of neutrophils. Prometryn treatment significantly increased the hepatic levels of 11-HETE and 15-HETE in this study. Notably, the levels of 11-HETE and 15-HETE were significantly increased in the liver of a mouse model of alcoholic liver disease^67^. Similarly, the hepatic concentrations of 11-HETE and 15-HETE were elevated in a CCl_4_-induced liver injury in mice. The study found that CCl_4_ activates AA metabolism and leads to increased M1-type macrophages and neutrophils and elevated cytokines, chemokines, and ROS levels in mice liver^68^. Serum/plasma concentrations of 11-HETE and 15-HETE were reported to be elevated in multiple studies of human patients with alcohol-related liver disease, nonalcoholic steatohepatitis, and nonalcoholic fatty liver disease^69–71^. LTB4 functions as a potent chemokine promoting migration of macrophages and neutrophils into tissues^72^. High-fat diet/obese mice exhibit increased levels of LTB4 (2–3 fold) in muscle, liver, and adipose tissue, compared to chow-fed lean mice^72^. The role of LTB4 is well characterized in contributing to hepatic ischemia–reperfusion injury in rats^73^, increasing inflammation and insulin resistance in hepatic cells^74^, and worsening severity of liver cirrhosis^75^. Likewise, we showed that prometryn significantly increased hepatic LTB4 levels in mice.

TriHOMEs have been reported to possess proinflammatory properties^76^ and to be dysregulated in respiratory disease, including asthma and chronic obstructive pulmonary disease^37,77,78^. 9,12,13-TriHOME was found elevated in prometryn treated mice liver. 9-HODE and 13-HODE are considered excellent biomarkers for lipid peroxidation^79^. 13-HODE has been reported to be enriched in oxidized low-density lipoproteins and may contribute to the accumulation of macrophages in atherosclerotic plaques suggesting a detrimental role for this oxylipin in atherosclerosis progression^76^. The levels of 9-HODE and 13-HODE were significantly increased in prometryn treated mice liver. Notably, Feldstein et al. observed that 9-HODE and 13-HODE were significantly elevated in patients with nonalcoholic steatohepatitis, inflammation and damage caused by a buildup of fat in the liver^39^. Previous studies have also implicated the overproduction of 9-HODE and 13-HODE in the progression of alcohol-induced liver injury in mice via the 12/15-LOX signaling pathway^80,81^. Atrazine, which belongs to the same class of pesticide as prometryn, increased the 9-HODE and 13-HODE levels^14^. Further investigations, both in vitro and in vivo, showed that increased oxidative stress is a core abnormality responsible for prometryn-induced liver toxicity in mice from our study. It also supports the hypothesis that pesticides exposure can contribute to disease progression like nonalcoholic fatty liver disease (NAFLD) and hepatocellular carcinoma if non-target organisms like humans are exposed for a long time via increase in oxidative stress, lipid peroxidation, and alterations in oxylipin metabolites profiles. It is possible that the underlying mechanisms by which prometryn exerts its deleterious effects in the liver is via the induction of pro-inflammatory response in macrophages by overproduction of 9-HODE and induction of oxidative stress and apoptosis in hepatocytes by overproduction of 13-HODE.

EpETrEs functions as autocrine and paracrine effectors in the cardiovascular and renal systems, which are believed to be largely beneficial. Because of the anti-hypertensive, fibrinolytic and anti-thrombotic properties of EpETrEs, their presence in red blood cells has important implications for the control of circulation and the physical properties of the circulating blood^82^. Plasma oxylipins were suggested as possible circulating biomarkers to detect cardiovascular dysfunctions or cardiovascular diseases (CVD)^83^. Caligiuri et al. showed that specific plasma oxylipins increased the odds of CVD and cerebrovascular events in patients with peripheral artery disease^84^. Our circulatory lipidomic profiling uncovered that prometryn treatment significantly decreased the level of 12(13)-EpODE, 15(16)-EpODE, 9(10)-EpODE, 14(15)-EpETE, 15-HEPE, 11(12)-EpETE, 17(18)-EpETE, 8(9)-EpETE, 8(9)-EpETrE, and 9,10,13-TriHOME in male mice liver. The lipidomic analysis showed that prometryn significantly decreased the level of EpFA synthesized by CYP, suggesting that the anti-hypertensive, fibrinolytic and anti-thrombotic properties of EETs were altered by prometryn.

## Conclusion

Persistent xenobiotics that are widely utilized in the environment, such as prometryn, can be toxic to the liver tissue. Prometryn hepatotoxic effects is probably due to diverse effects on the liver lipid metabolism, including induction of lipid peroxidation, oxidative stress, endoplasmic reticulum stress, activation of apoptotic pathway, increased sEH and mEH expression and enzymatic activities, inflammatory responses, and alterations of oxylipin metabolites.

### Supplementary Information

Supplementary Figures.

Supplementary Tables 1 and 2

Supplementary Table 3.

Supplementary Table 4.

Supplementary Table 5.

Supplementary Table 6.

## Data Availability

The lipidomic dataset is included as Supplementary Table 5. Any other datasets used and/or analysed during the current study are available from the corresponding author upon reasonable request.
