# Supplementary material for: Triazine herbicide prometryn alters epoxide hydrolase activity and increases cytochrome P450 metabolites in murine livers via lipidomic profiling
Source: Sci Rep. 2024 Aug 19;14:19135. doi: 10.1038/s41598-024-69557-3 (PMC11333623; doi:10.1038/s41598-024-69557-3)
Supplement: Supplementary file 1 — Supplementary Figures. [file 41598_2024_69557_MOESM1_ESM.pdf]

Supplementary Information

Triazine herbicide prometryn alters epoxide hydrolase activity and increases cytochrome P450 metabolites in murine livers via lipidomic profiling

Rasheed O. Sule, Jun Yang, Christophe Morisseau, Bruce Hammock, and Aldrin V. Gomes

Supplementary Figures:

Supplementary Figure 1. Prometryn increased the level of a pro-inflammatory mediator (Leukotriene B4/LTB4). (a) Plot showing that prometryn treatment increased the hepatic concentration of LOX-derived oxylipin through the AA pathway. Bars represent the mean ± SEM; n = 10 to 11 mice per group. $*p < 0.05$.

Supplementary Figure 2. Proteasome and Immunoproteasome activities in prometryn treated mice. (a) Prometryn treatment significantly increased the β1 and β2 proteasome subunit activities and decreased the β5 proteasome activity. (b) Prometryn treatment did not significantly affect immunoproteasome activities.

Supplementary Figure 3. Raw spectral data for the oxylipins (a) 14,15 EpETrE, (b) 12,13 DiHOME, (c) 14,15 DiHETrE measured in standard (left side) and liver sample (right right) extraction.

Supplementary Figure 4-6. Uncropped western blots of the blots presented in the manuscript. Blots were cut into strips and then the Western blotting procedure as detailed in method section was carried out.

Supplementary Tables:

Supplementary Table 1. Table showing the concentration of all detected oxylipin metabolites in liver tissues of mice treated with vehicle control and prometryn.

Supplementary Table 2. Table showing the concentration of all detected oxylipin metabolites in plasma of mice treated with vehicle control and prometryn.

Supplementary Table 3. Excel file showing the Limit Of Quantification (LOQ) for the oxylipins detected.

Supplementary Table 4. Excel file showing the concentration of all detected oxylipin metabolites in the corn oil sample used as the vehicle.

Supplementary Table 5. Excel file showing the raw liver and plasma oxylipin results.

Supplementary Table 6. Excel file showing the reproducibility of the same sample of liver analyzed for oxylipins.

(a)

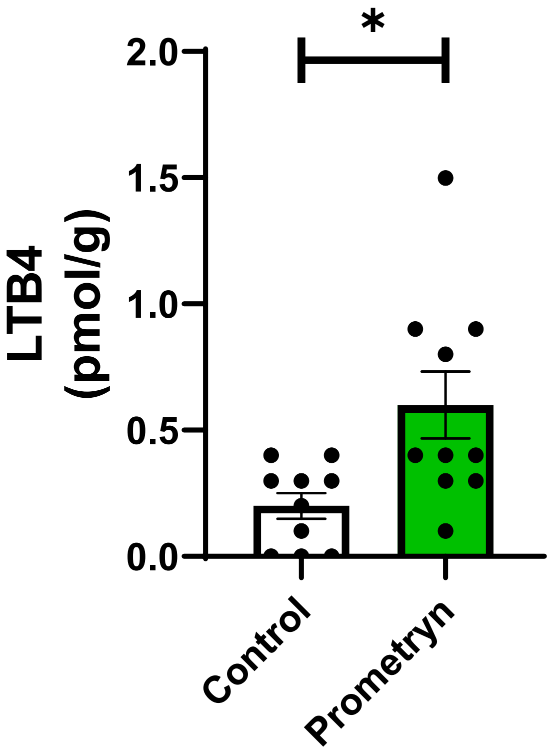

Supplementary Figure 1.

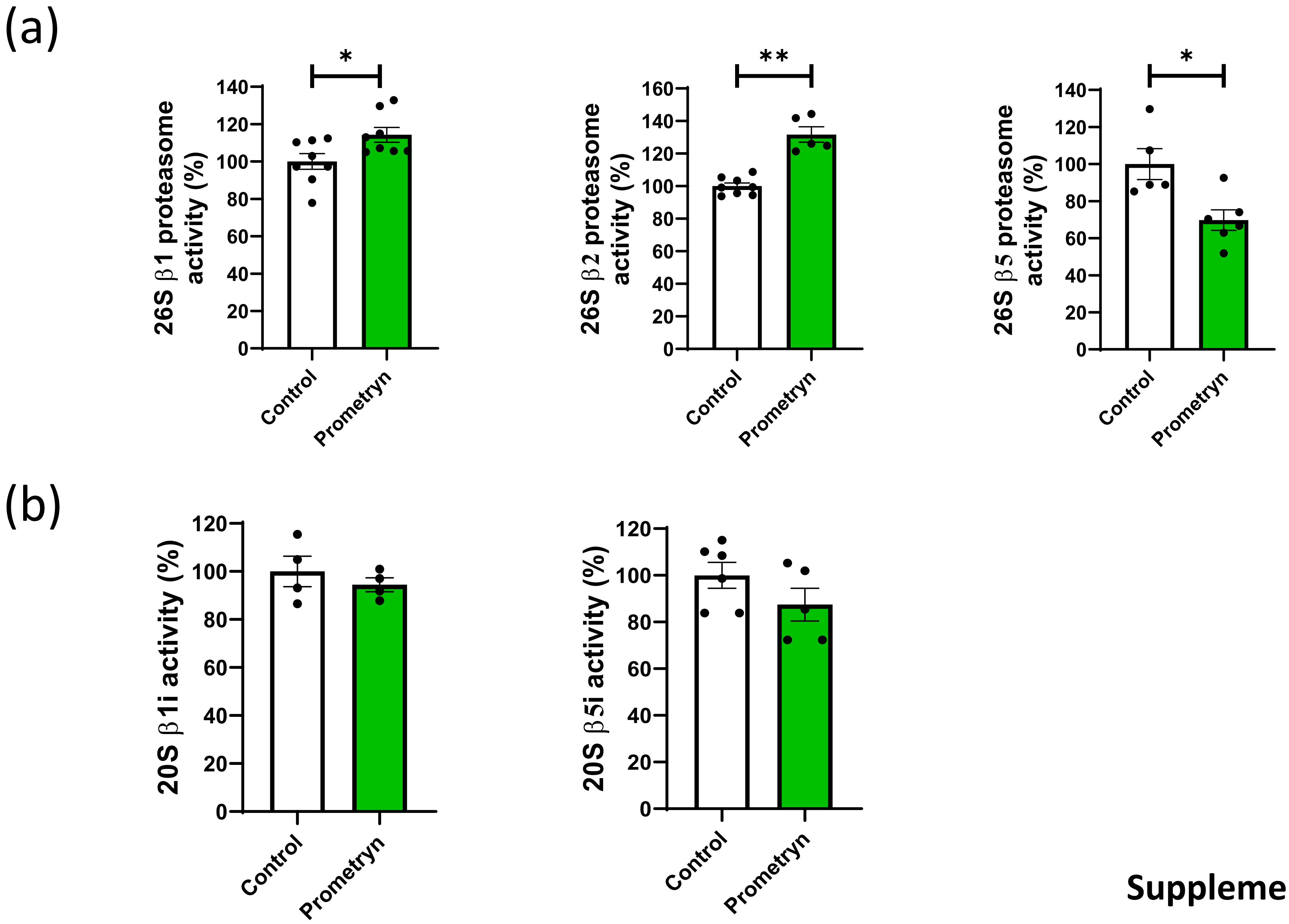

Supplementary Figure 2.

(a) 14,15 EpETrE

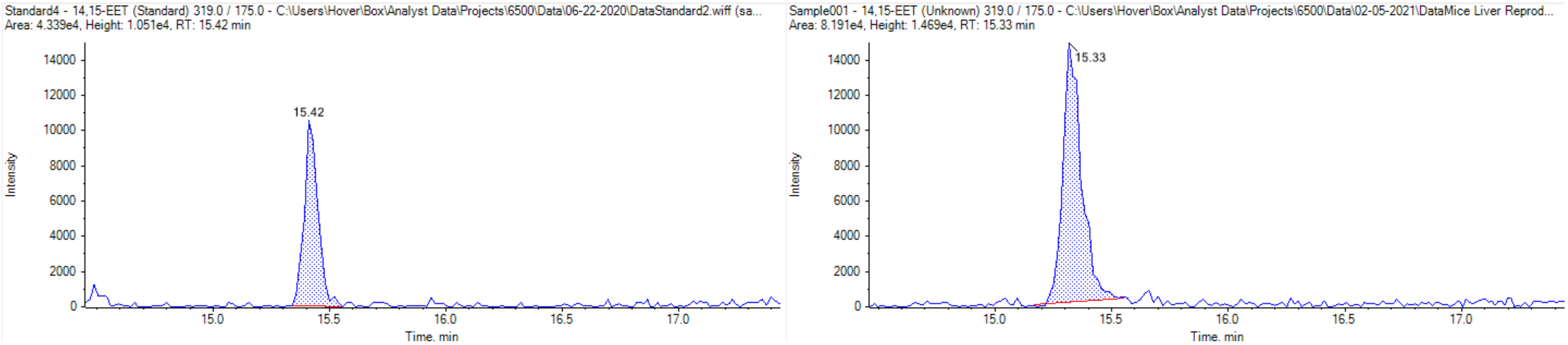

(b) 12,13 DiHOME

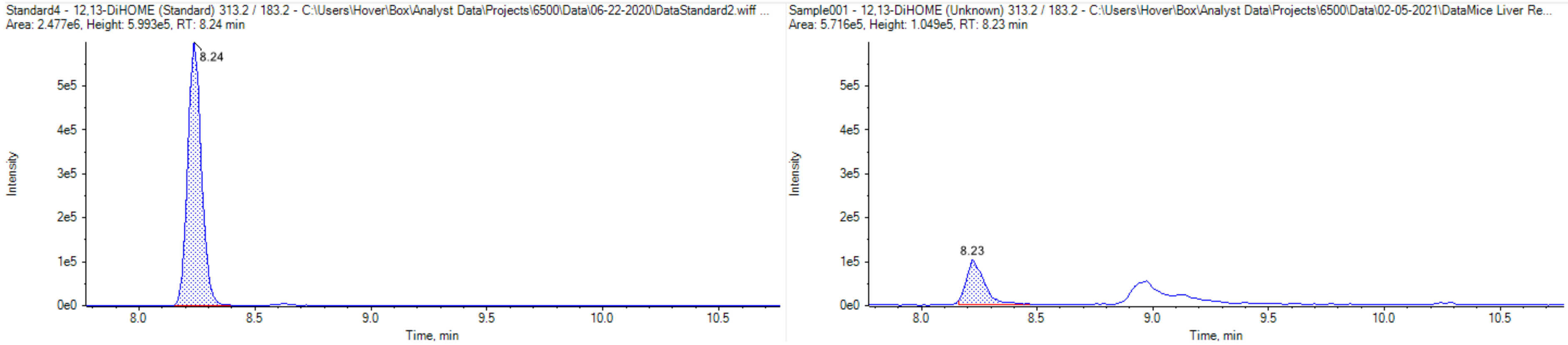

Supplementary Figure 3.

(c) 14,15 DiHETrE

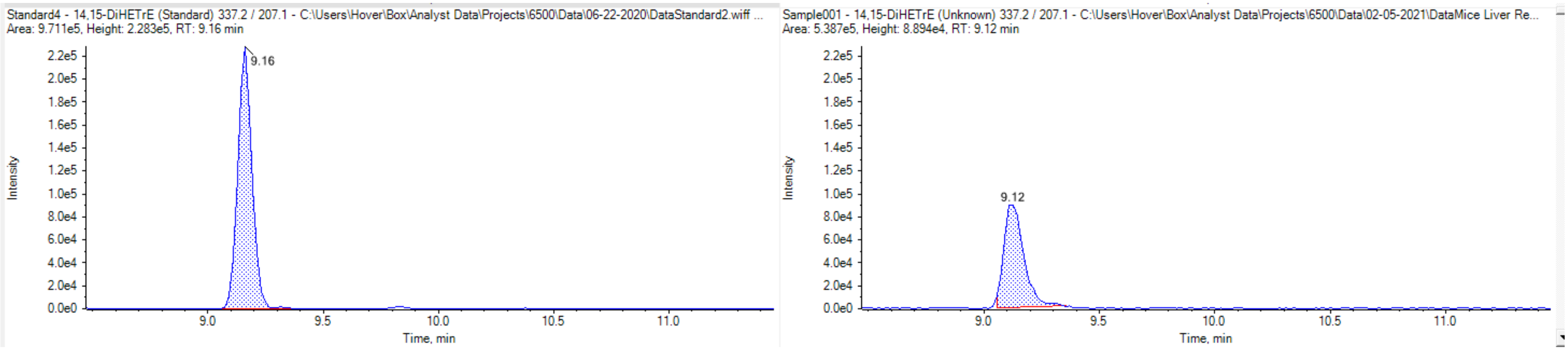

Supplementary Figure 3.

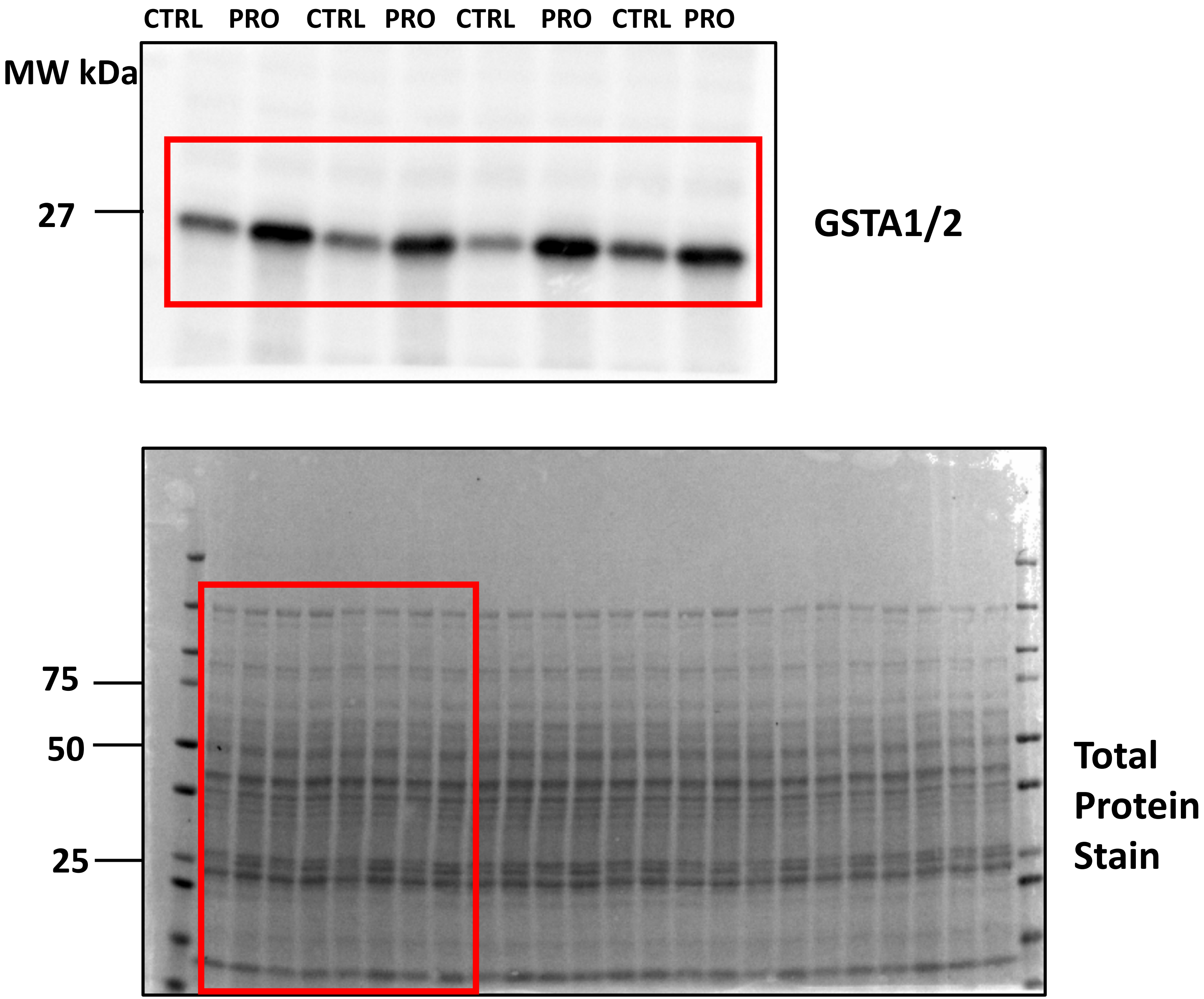

Supplementary Figure 4.

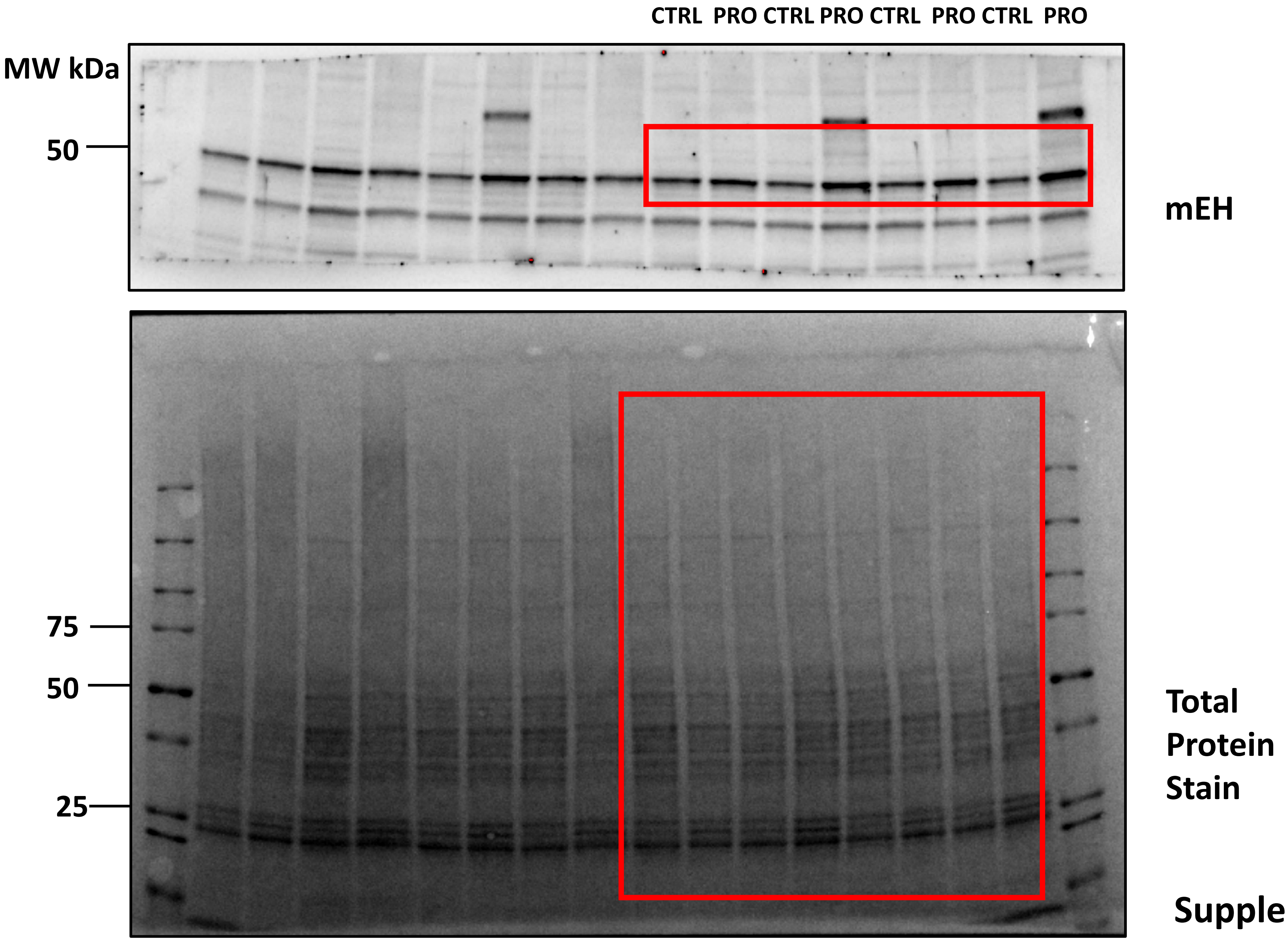

Supplementary Figure 5.

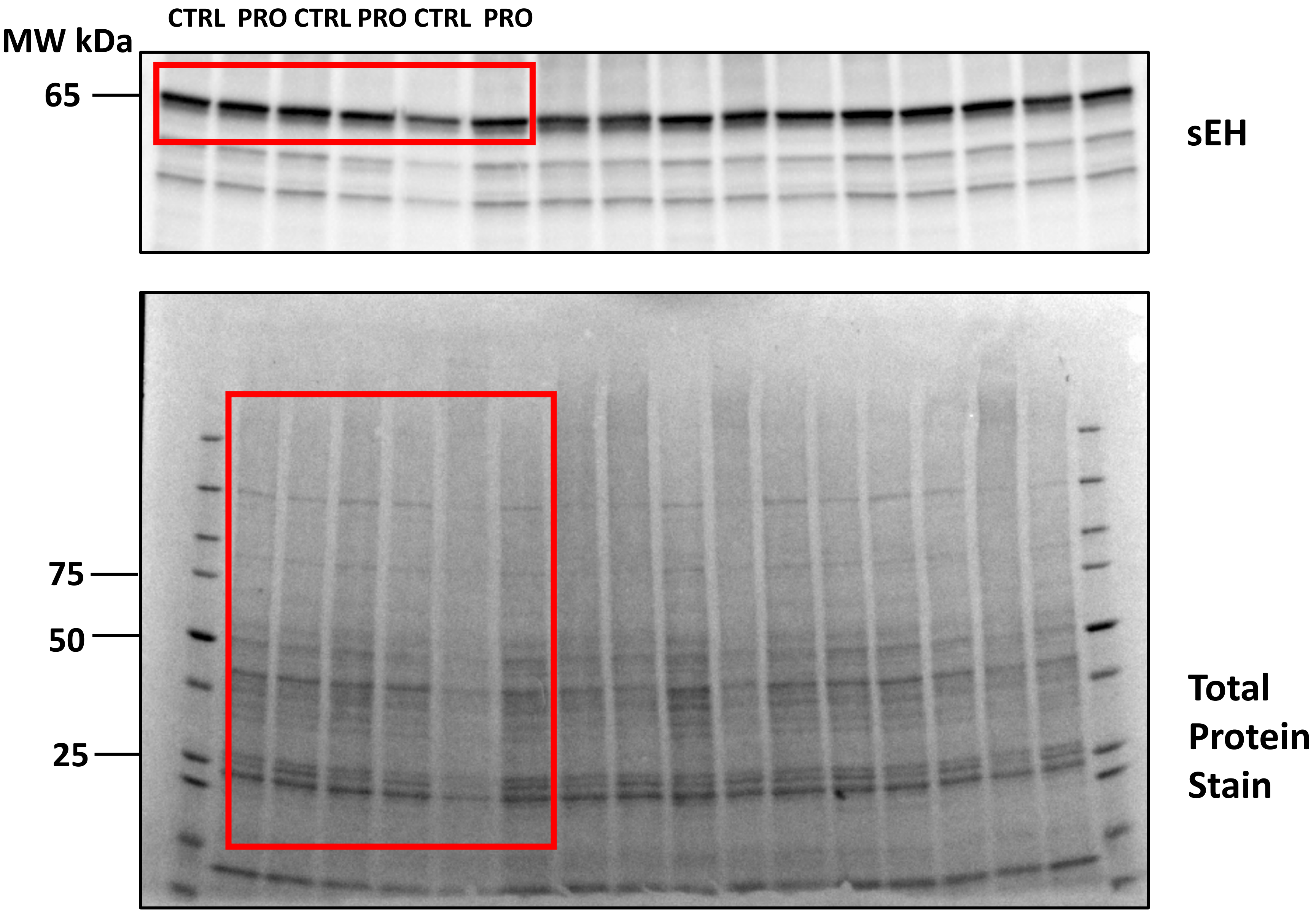

Supplementary Figure 6.
