# Supplementary material for: Triazine herbicide prometryn alters epoxide hydrolase activity and increases cytochrome P450 metabolites in murine livers via lipidomic profiling
Source: Sci Rep. 2024 Aug 19;14:19135. doi: 10.1038/s41598-024-69557-3 (PMC11333623; doi:10.1038/s41598-024-69557-3)
Supplement: Supplementary file 2 — Supplementary Tables 1 and 2 [file 41598_2024_69557_MOESM2_ESM.docx]

**Supplementary Table 1.** Table showing the concentration of all detected oxylipin metabolites in liver tissues of mice treated with vehicle control and prometryn.

| Oxylipins | Control | | Prometryn | | Significance | |
| --- | --- | --- | --- | --- | --- | --- |
|  | Mean ± SD  (pmol/g) | n | Mean ± SD  (pmol/g) | n | p-value | * |
| 12(13)-EpODE | 1.5665 ± 0.02 | 2 | 1.6273 ± 1.67 | 8 | 0.962 | no |
| 9-HOTrE | 70.0761 ± 43.36 | 10 | 87.6905 ± 32.40 | 11 | 0.302 | no |
| 13-HOTrE | 119.8526 ± 64.28 | 10 | 105.1384 ± 37.17 | 11 | 0.523 | no |
| **15(16)-EpODE** | **27.6128 ± 32.63** | **10** | **129.8329 ± 99.83** | **11** | **0.006** | **Y** |
| 9(10)-EpODE | 5.3929 ± 7.18 | 10 | 15.4597 ± 14.90 | 11 | 0.270 | no |
| **13-HODE** | **76.1712 ± 30.70** | **10** | **115.1295 ± 48.27** | **11** | **0.042** | **Y** |
| **9-HODE** | **74.205 ± 32.64** | **10** | **114.3237 ± 45.20** | **11** | **0.032** | **Y** |
| 12(13)-EpOME | 8.0477 ± 10.35 | 6 | 33.0609 ± 0.00 | 1 | n/a | n/a |
| 9(10)-EpOME | 3.2637 ± 3.54 | 8 | 11.5974 ± 8.20 | 4 | 0.1311 | no |
| 12,13-DiHODE | 5.0115 ± 8.57 | 9 | 0.5171 ± 0.46 | 11 | 0.098 | no |
| **15,16-DiHODE** | **1.8544 ± 0.68** | **10** | **3.549 ± 1.95** | **11** | **0.017** | **Y** |
| **9,10-DiHODE** | **0.228 ± 0.08** | **10** | **0.516 ± 0.32** | **11** | **0.012** | **Y** |
| 12,13-DiHOME | 6.6165 ± 1.86 | 10 | 9.4014 ± 4.03 | 11 | 0.06 | no |
| 9,10-DiHOME | 2.5739 ± 0.91 | 10 | 3.1276 ± 1.47 | 11 | 0.317 | no |
| 11-HEPE | 37.1944 ± 24.87 | 8 | 24.2119 ± 9.08 | 9 | 0.164 | no |
| 12-HEPE | 45.5479 ± 45.15 | 10 | 36.7318 ± 15.44 | 11 | 0.549 | no |
| **14(15)-EpETE** | **1.8324 ± 2.32** | **10** | **8.0613 ± 4.38** | **11** | **<0.001** | **Y** |
| 15-HEPE | 2.9449 ± 1.83 | 10 | 3.1992 ± 1.11 | 11 | 0.702 | No |
| **11(12)-EpETE** | **2.7355 ± 3.58** | **10** | **11.9290 ± 5.86** | **11** | **<0.001** | **Y** |
| 15-oxo-ETE | 6.8711 ± 2.88 | 8 | 4.736 ± 1.49 | 11 | 0.0866 | No |
| 17(18)-EpETE | 10.0784 ± 12.82 | 6 | 21.7977 ± 8.77 | 7 | 0.077 | No |
| 5-HEPE | 4.2287 ± 2.08 | 10 | 3.6265 ± 1.07 | 11 | 0.409 | No |
| **8(9)-EpETE** | **6.409 ± 9.76** | **10** | **29.3083 ± 15.63** | **11** | **<0.001** | **Y** |
| **8-HEPE** | **2.4612 ± 2.03** | **10** | **0.9093 ± 0.68** | **11** | **0.021** | **Y** |
| 9-HEPE | 30.8141 ± 20.46 | 6 | 26.6684 ± 13.62 | 8 | 0.656 | No |
| **11(12)-EpETrE** | **4.65 ± 2.49** | **8** | **12.1828 ± 4.45** | **6** | **0.0067** | **Y** |
| **11-HETE** | **22.7672 ± 10.49** | **10** | **41.6532 ± 17.89** | **11** | **0.009** | **Y** |
| 12-HETE | 7.6626 ± 8.42 | 10 | 6.8206 ± 2.97 | 11 | 0.759 | No |
| **14(15)-EET** | **4.5362 ± 5.58** | **10** | **14.0181 ± 7.84** | **11** | **0.005** | **Y** |
| **15-HETE** | **21.0699 ± 9.46** | **10** | **40.4254 ± 19.75** | **11** | **0.011** | **Y** |
| **5-HETE** | **1.2577 ± 0.68** | **10** | **0.6523 ± 0.23** | **11** | **0.011** | **Y** |
| **8(9)-EpETrE** | **4.0933 ± 3.68** | **7** | **38.3601 ± 22.55** | **9** | **0.0017** | **Y** |
| 8-HETE | 0.5928 ± 0.37 | 9 | 1.0253 ± 0.65 | 11 | 0.092 | No |
| 9-HETE | 1.2077 ± 0.88 | 10 | 1.0731 ± 0.44 | 11 | 0.657 | No |
| 15(S)-HETrE | 11.3821 ± 7.29 | 10 | 12.3066 ± 5.5743 | 11 | 0.746 | No |
| 15-oxoEDE | 21.6392 ± 9.98 | 8 | 13.2849 ± 10.8492 | 9 | 0.121 | No |
| **5-HETrE** | **10.1176 ± 7.89** | **8** | **2.814 ± 2.0414** | **8** | **0.0354** | **Y** |
| 8-HETrE | ND | ND | ND | ND | ND | ND |
| 9,10,13-TriHOME | 32.8511 ± 23.22 | 10 | 55.5635 ± 30.3536 | 11 | 0.071 | No |
| **9,12,13-TriHOME** | **27.2856 ± 18.61** | **10** | **52.5975 ± 28.8429** | **11** | **0.029** | **Y** |
| **11,12-DiHETE** | **5.5013 ± 1.45** | **10** | **9.8157 ± 3.1045** | **11** | **<0.001** | **Y** |
| **14,15-DiHETE** | **15.4618 ± 3.73** | **10** | **20.7544 ± 6.9207** | **11** | **0.045** | **Y** |
| **17,18-DiHETE** | **28.2862 ± 3.87** | **10** | **44.3718 ± 14.2813** | **11** | **0.003** | **Y** |
| 5,15-DiHETE | 7.5976 ± 7.98 | 5 | 5.1271 ± 2.9949 | 11 | 0.371 | no |
| 5,6-DiHETE | 0.3379 ± 0.20 | 10 | 0.1943 ± 0.1122 | 10 | 0.061 | no |
| 6-trans-LTB4 | 0.8025 ± 0.66 | 3 | 0.3205 ± 0.3066 | 7 | 0.139 | no |
| **8,15-DiHETE** | **23.3316 ±12.89** | **10** | **38.0209 ± 14.7861** | **11** | **0.026** | **Y** |
| **8,9-DiHETE** | **3.0319 ± 0.94** | **10** | **4.2204 ± 1.1312** | **11** | **0.018** | **Y** |
| **LTB4** | **0.2854 ± 0.1069** | **7** | **0.6000 ± 0.411** | **10** | **0.0452** | **Y** |
| 11,12-DiHETrE | 7.1911 ± 2.39 | 10 | 9.2596 ± 2.917 | 11 | 0.093 | no |
| **14,15-DiHETrE** | **16.8244 ± 3.80** | **10** | **27.4922 ± 6.1786** | **11** | **<0.001** | **Y** |
| 5,6-DiHETrE | 0.783 ± 0.29 | 10 | 0.8095 ± 0.3375 | 11 | 0.849 | no |
| 8,9-DiHETrE | 2.8121 ± 1.14 | 10 | 2.8782 ± 0.8733 | 11 | 0.882 | no |
| 10(11)-EpDPE | 10.336 ± 5.19 | 7 | 25.6152 ± 6.551 | 2 | 0.1425 | no |
| 13(14)-EpDPE | 4.802 ± 3.19 | 10 | 6.8155 ± 3.9103 | 9 | 0.234 | no |
| **16(17)-EpDPE** | **5.2652 ± 3.13** | **9** | **13.2876 ± 5.2723** | **6** | **0.0113** | **Y** |
| **19(20)-EpDPE** | **17.8289 ± 24.40** | **10** | **47.5118 ± 18.2168** | **11** | **0.005** | **Y** |
| 7(8)-EpDPE | 323.1366 ± 253.34 | 7 | 1633.4003 ± 925.23 | 3 | 0.1295 | No |
| 20-OH-LTB4 | 0.3031 ± 0.11 | 4 | 2.9893 ± 7.3906 | 7 | 0.496 | no |
| PGD2 | 6.132 ± 6.40 | 9 | 6.8434 ± 4.096 | 11 | 0.766 | no |
| **PGE2** | **3.0808 ± 3.23** | **9** | **0.3898 ± 0.2403** | **10** | **0.0363** | **Y** |
| **11,12-,15-TriHETrE** | **67.8234 ± 40.46** | **10** | **284.7188 ± 159.0293** | **11** | **<0.001** | **Y** |
| PGD1 | 1.1916 ± 0.83 | 8 | 1.3353 ± 2.0516 | 4 | 0.863 | no |
| PGE1 | 5.7932 ± 1.10 | 10 | 5.5716 ± 0.7081 | 11 | 0.585 | no |
| PGF2a | 3.1084 ± 2.01 | 10 | 2.8992 ± 1.3243 | 11 | 0.779 | no |
| 10,11-DiHDPE | 7.0198 ± 2.73 | 10 | 7.3688 ± 2.4184 | 11 | 0.759 | no |
| 13,14-DiHDPE | 8.8117 ± 3.44 | 10 | 8.8946 ± 2.3277 | 11 | 0.949 | no |
| 16,17-DiHDPE | 28.3286 ± 9.46 | 10 | 29.0666 ± 7.2397 | 11 | 0.842 | no |
| 19,20-DiHDPE | 85.6109 ± 25.12 | 10 | 101.2834 ± 21.7544 | 11 | 0.142 | no |
| 4,5-DiHDPE | 1.5822 ± 0.67 | 7 | 1.3805 ± 0.6872 | 9 | 0.566 | no |
| 7,8-DiHDPE | 1.5822 ± 1.77 | 10 | 1.3805 ± 1.3215 | 11 | 0.965 | no |
| **20-COOH-LTB4** | **7.4184 ± 4.26** | **6** | **21.8869 ± 4.1347** | **2** | **0.006** | **Y** |
| TXB2 | 3.8515 ± 0.49 | 8 | 4.0883 ± 0.6255 | 10 | 0.395 | no |

**Supplementary Table 2.** Table showing the concentration of all detected oxylipin metabolites in plasma of mice treated with vehicle control and prometryn.

| Oxylipins | Control | | Prometryn | | Significance | |
| --- | --- | --- | --- | --- | --- | --- |
|  | Mean ± SD  (nmol/L) | N | Mean ± SD  (nmol/L) | n | p-value | * |
| **12(13)-EpODE** | **120.59 ± 86.09** | **8** | **47.57 ± 53.31** | **11** | **0.035** | **Y** |
| 13-oxo-ODE | 3384.19 ± 2999.68 | 8 | 2715.18 ± 2215.49 | 11 | 0.582 | no |
| 9-HOTrE | 2177.14 ± 1663.76 | 8 | 1921.21 ± 1247.18 | 11 | 0.706 | no |
| 13-HOTrE | 6218.05 ± 4176.25 | 8 | 6023.56 ± 3121.95 | 11 | 0.909 | no |
| **15(16)-EpODE** | **3177.93 ± 2557.68** | **8** | **1136.46 ± 1521.93** | **11** | **0.043** | **Y** |
| **9(10)-EpODE** | **1016.81 ± 649.32** | **8** | **382.99 ± 371.97** | **11** | **0.015** | **Y** |
| 13-HODE | 3861.20 ± 2276.71 | 8 | 3400.65 ± 1714.54 | 11 | 0.621 | no |
| 9-HODE | 2498.83 ± 1783.31 | 8 | 1899.50 ± 1207.11 | 11 | 0.393 | no |
| 12(13)-EpOME | 919.08 ± 484.05 | 8 | 834.64 ± 918.82 | 11 | 0.816 | no |
| 9(10)-EpOME | 891.95 ± 467.60 | 8 | 876.11 ± 942.64 | 11 | 0.966 | no |
| EKODE | 27232.10 ± 13631.44 | 8 | 11366.60 ± 12945.60 | 11 | 0.019 | no |
| 12,13-DiHODE | 9.61 ± 8.12 | 8 | 7.77 ± 7.51 | 11 | 0.617 | no |
| 15,16-DiHODE | 25.82 ± 28.37 | 8 | 23.12 ± 26.65 | 11 | 0.834 | no |
| 9,10-DiHODE | 10.58 ± 7.93 | 8 | 13.97 ± 18.60 | 11 | 0.636 | no |
| 12,13-DiHOME | 77.90 ± 53.00 | 8 | 73.91 ± 73.13 | 11 | 0.897 | no |
| 9,10-DiHOME | 48.95 ± 24.99 | 8 | 62.13 ± 78.11 | 11 | 0.653 | no |
| 11-HEPE | 226.65 ± 109.16 | 8 | 215.54 ± 259.56 | 11 | 0.911 | no |
| 12-HEPE | 2884.78 ± 2404.20 | 8 | 1988.41 ± 2637.56 | 11 | 0.459 | no |
| **14(15)-EpETE** | **52.05 ± 33.63** | **8** | **17.90 ± 18.31** | **11** | **0.011** | **Y** |
| **15-HEPE** | **67.90 ± 38.78** | **8** | **39.42 ± 17.50** | **11** | **0.045** | **Y** |
| **11(12)-EpETE** | **54.44 ± 34.54** | **8** | **19.53 ± 20.46** | **11** | **0.013** | **Y** |
| 15-oxo-ETE | 236.91 ± 165.55 | 8 | 167.29 ± 104.59 | 11 | 0.276 | no |
| **17(18)-EpETE** | **92.70 ± 64.40** | **8** | **28.77 ± 27.10** | **11** | **0.009** | **Y** |
| 5-HEPE | 90.80 ± 58.73 | 8 | 93.94 ± 70.03 | 11 | 0.919 | no |
| **8(9)-EpETE** | **102.88 ± 62.89** | **8** | **36.52 ± 34.23** | **11** | **0.009** | **Y** |
| 8-HEPE | 37.51 ± 17.19 | 8 | 43.13 ± 53.94 | 11 | 0.781 | no |
| 9-HEPE | 302.07 ± 122.77 | 8 | 318.59 ± 378.48 | 11 | 0.907 | no |
| 11(12)-EpETrE | 223.99 ± 137.27 | 8 | 114.64 ± 131.63 | 11 | 0.097 | no |
| 11-HETE | 953.76 ± 578.14 | 8 | 883.71 ± 796.31 | 11 | 0.835 | no |
| 12-HETE | 2005.81 ± 1401.05 | 8 | 2014.69 ± 2658.03 | 11 | 0.993 | no |
| 14(15)-EpETrE | 230.68 ± 150.70 | 8 | 141.03 ± 153.50 | 11 | 0.222 | no |
| 15-HETE | 619.98 ± 380.49 | 8 | 502.42 ± 408.18 | 11 | 0.532 | no |
| 5-HETE | 42.04 ± 27.90 | 8 | 58.15 ± 46.42 | 11 | 0.396 | no |
| **8(9)-EpETrE** | **304.53 ± 285.84** | **8** | **58.90 ± 51.90** | **11** | **0.012** | **Y** |
| 8-HETE | 49.12 ± 31.35 | 8 | 41.51 ± 30.60 | 11 | 0.603 | no |
| 9-HETE | 60.97 ± 57.34 | 8 | 36.49 ± 22.43 | 11 | 0.212 | no |
| 15(S)-HETrE | 201.37 ± 154.42 | 8 | 99.30 ± 50.77 | 11 | 0.055 | no |
| 15-oxoEDE | 442.37 ± 348.74 | 8 | 226.43 ± 108.72 | 11 | 0.068 | no |
| 5-HETrE | 178.13 ± 161.08 | 8 | 171.82 ± 128.42 | 11 | 0.925 | no |
| **9,10,13-TriHOME** | **3233.00 ± 1848.24** | **8** | **1569.55 ± 677.68** | **11** | **0.013** | **Y** |
| 9,12,13-TriHOME | 2733.67 ± 1683.33 | 8 | 1580.62 ± 793.32 | 11 | 0.062 | no |
| LTB5 | 7.01 ± 4.71 | 8 | 8.83 ± 6.95 | 11 | 0.53 | no |
| 11,12-DiHETE | 4.53 ± 3.10 | 8 | 5.09 ± 3.56 | 11 | 0.727 | no |
| 14,15-DiHETE | 9.61 ± 8.88 | 8 | 7.83 ± 8.99 | 11 | 0.674 | no |
| 17,18-DiHETE | 16.05 ± 20.02 | 8 | 12.03 ± 14.78 | 11 | 0.62 | no |
| 5,15-DiHETE | 26.20 ± 9.75 | 6 | 22.44 ± 7.73 | 6 | 0.477 | no |
| 5,6-DiHETE | 4.38 ± 3.38 | 8 | 5.54 ± 4.03 | 11 | 0.518 | no |
| 6-trans-LTB4 | 22.87 ± 15.34 | 8 | 31.65 ± 21.93 | 11 | 0.346 | no |
| 8,15-DiHETE | 190.94 ± 99.81 | 8 | 178.81 ± 120.37 | 11 | 0.819 | no |
| 8,9-DiHETE | 12.79 ± 9.53 | 8 | 8.61 ± 7.56 | 11 | 0.301 | no |
| LTB4 | 18.32 ± 8.19 | 8 | 15.92 ± 9.52 | 11 | 0.573 | no |
| 11,12-DiHETrE | 6.05 ± 5.25 | 8 | 3.85 ± 3.35 | 11 | 0.28 | no |
| 14,15-DiHETrE | 10.79 ± 8.56 | 8 | 6.11 ± 4.88 | 11 | 0.148 | no |
| 5,6-DiHETrE | 7.55 ± 7.39 | 8 | 8.39 ± 7.75 | 11 | 0.817 | no |
| 8,9-DiHETrE | 4.52 ± 3.30 | 8 | 4.14 ± 3.87 | 11 | 0.825 | no |
| 10(11)-EpDPE | 366.16 ± 266.36 | 8 | 261.89 ± 338.21 | 11 | 0.48 | no |
| 13(14)-EpDPE | 150.71 ± 98.20 | 8 | 173.70 ± 242.25 | 11 | 0.804 | no |
| 16(17)-EpDPE | 119.80 ±70.69 | 8 | 150.71 ± 219.31 | 11 | 0.707 | no |
| 19(20)-EpDPE | 518.55 ± 256.96 | 8 | 282.98 ± 257.11 | 11 | 0.065 | no |
| **7(8)-EpDPE** | **8146.75 ± 7102.16** | **7** | **1132.57 ± 1116.06** | **9** | **0.011** | **Y** |
| 20-OH-LTB4 | 1.26 ± 0.70 | 5 | 0.89 ± 0.76 | 9 | 0.389 | no |
| PGD2 | 47.72 ± 15.67 | 8 | 60.51 ± 41.55 | 11 | 0.422 | no |
| PGE2 | 27.34 ± 20.18 | 8 | 17.47 ± 12.54 | 11 | 0.205 | no |
| 11,12-,15-TriHETrE | 13787.64 ± 8994.98 | 8 | 7863.01 ± 4682.50 | 11 | 0.078 | no |
| PGD1 | 27.96 ± 12.34 | 8 | 34.01 ± 19.49 | 11 | 0.452 | no |
| PGE1 | 42.81 ± 15.14 | 8 | 40.13 ± 23.12 | 11 | 0.779 | no |
| PGF2a | 11.19 ± 6.19 | 8 | 8.63 ± 7.63 | 11 | 0.448 | no |
| 10,11-DiHDPE | 11.36 ± 9.24 | 8 | 7.21 ± 5.39 | 11 | 0.234 | no |
| 13,14-DiHDPE | 8.78 ± 8.35 | 8 | 5.26 ± 5.48 | 11 | 0.281 | no |
| 16,17-DiHDPE | 29.01 ± 25.10 | 8 | 21.42 ± 21.72 | 11 | 0.49 | no |
| 19,20-DiHDPE | 77.83 ± 81.49 | 8 | 49.67 ± 59.69 | 11 | 0.395 | no |
| 4,5-DiHDPE | 3.27 ± 1.02 | 4 | 5.15 ± 6.25 | 6 | 0.575 | no |
| 7,8-DiHDPE | 13.69 ± 8.99 | 8 | 15.52 ± 15.00 | 11 | 0.763 | no |
| 20-COOH-LTB4 | 59.49 ± 20.07 | 8 | 56.15 ± 30.33 | 10 | 0.793 | no |
| TXB2 | 41.70 ± 25.95 | 8 | 57.79 ± 35.09 | 11 | 0.289 | no |
| 6-keto-PGF1a | 952214.22 ± 980125.97 | 8 | 682207.13 ±  600380.74 | 11 | 0.466 | no |

Statistical significance was determined using unpaired two-tailed Student’s t-test between the control and prometryn group. **Bold values represent oxylipins, which were found to be significant in both liver tissue and plasma.**
